# Variation in One Residue Associated with the Metal Ion-Dependent Adhesion Site Regulates αIIbβ3 Integrin Ligand Binding Affinity

**DOI:** 10.1371/journal.pone.0076793

**Published:** 2013-10-08

**Authors:** Joel Raborn, Ting Fu, Xue Wu, Zhilong Xiu, Guohui Li, Bing-Hao Luo

**Affiliations:** 1 Department of Biological Sciences, Louisiana State University, Baton Rouge, Louisiana, United States of America; 2 Laboratory of Molecular Modeling and Design, State Key Laboratory of Molecular Reaction Dynamics, Dalian Institute of Chemical Physics, Chinese Academy of Sciences, Dalian, PR China; 3 Department of Bioscience and Biotechnology, Dalian University of Technology, Dalian, PR China; 4 Graduate University of the Chinese Academy of Sciences, Beijing, P. R. China; 5 University of Alabama at Birmingham School of Medicine, Birmingham, Alabama, United States of America; Dresden University of Technology, Germany

## Abstract

The Asp of the RGD motif of the ligand coordinates with the β I domain metal ion dependent adhesion site (MIDAS) divalent cation, emphasizing the importance of the MIDAS in ligand binding. There appears to be two distinct groups of integrins that differ in their ligand binding affinity and adhesion ability. These differences may be due to a specific residue associated with the MIDAS, particularly the β3 residue Ala^252^ and corresponding Ala in the β1 integrin compared to the analogous Asp residue in the β2 and β7 integrins. Interestingly, mutations in the adjacent to MIDAS (ADMIDAS) of integrins α4β7 and αLβ2 increased the binding and adhesion abilities compared to the wild-type, while the same mutations in the α2β1, α5β1, αVβ3, and αIIbβ3 integrins demonstrated decreased ligand binding and adhesion. We introduced a mutation in the αIIbβ3 to convert this MIDAS associated Ala^252^ to Asp. By combination of this mutant with mutations of one or two ADMIDAS residues, we studied the effects of this residue on ligand binding and adhesion. Then, we performed molecular dynamics simulations on the wild-type and mutant αIIbβ3 integrin β I domains, and investigated the dynamics of metal ion binding sites in different integrin-RGD complexes. We found that the tendency of calculated binding free energies was in excellent agreement with the experimental results, suggesting that the variation in this MIDAS associated residue accounts for the differences in ligand binding and adhesion among different integrins, and it accounts for the conflicting results of ADMIDAS mutations within different integrins. This study sheds more light on the role of the MIDAS associated residue pertaining to ligand binding and adhesion and suggests that this residue may play a pivotal role in integrin-mediated cell rolling and firm adhesion.

## Introduction

Integrins are cell adhesion molecules that transmit bidirectional signals across the plasma membrane [1,2]. Divalent metal ions regulate integrin ligand binding and signaling. Specifically, Ca^2+^ stabilizes the low affinity state, while some other ions such as Mn^2+^ promote ligand binding [3,4,5,6]. Three metal ion binding sites are present in the β subunit I domain, which have been shown to account for this regulation. The metal ion-dependent adhesion site (MIDAS) is located in the center, flanked by two allosteric sites, named the synergistic metal binding site (SyMBS) and the adjacent to MIDAS (ADMIDAS) on either side [4,6,7,8,9,10,11,12,13,14,15,16,17].

The MIDAS is essential for ligand binding. In normal physiological conditions, the MIDAS residues, including the conservative DXSXS motif, coordinate with Mg^2+^ [4,6,7,9,12,14,15]. The carboxyl group of the Asp in the RGD motif of the ligand has been shown to associate with the β3 I MIDAS Mg^2+^ during ligand binding [13,16]. Previous studies have shown that mutations in the MIDAS abolished ligand binding, underscoring its importance for ligand binding and subsequent signaling [4,8,18,19,20]. Adjoining the MIDAS, the SyMBS coordinates Ca^2+^ and stabilizes the MIDAS ion for ligand binding, subsequently acting as an allosteric activator. In low concentrations of Ca^2+^, the SyMBS coordinates with low concentrations of MIDAS Mg^2+^ to facilitate ligand binding [10,13,17]. The SyMBS has been described through mutagenesis studies as important but not required for ligand binding, especially if the integrin is in the extended open headpiece confirmation. Although several properties of the SyMBS have been elucidated, the exact functional role of this metal ion-binding site remains elusive [4,6,20,21,22,23,24,25]. The third ion-binding site is located on the opposite side of the MIDAS from the SyMBS and coordinates Ca^2+^ [4,14,20,21]. In the unliganded closed headpiece crystal structure of the αIIbβ3 [17], the ADMIDAS Ca^2+^ ion associates with the carbonyl backbone of Met^335^. Integrin inside-out activation causes the β3 hybrid domain to swing out, followed by the downward displacement of the β3 I domain α7 helix and thus breaking the bond between Met^335^ and the ADMIDAS ion [16,21,26,27,28,29]. Following these conformational changes, the metal ion-binding sites also change conformation, particularly the Asp^251^ side chain carboxyl moves toward the ADMIDAS site and coordinates with its Ca^2+^ ion. Because these residue shifts, the αIIbβ3 integrin is now in the high affinity open headpiece conformation [16], making the MIDAS become more positive and able to bind ligands with higher affinity [17]. Thus, the ADMIDAS ion not only stabilizes the low affinity closed headpiece conformation but also stabilizes the high affinity state with the open headpiece conformation, which has been confirmed by mutagenesis studies [17,25].

As previously reported, differences exist in the ligand binding affinity and adhesion ability among integrins [4,6,14,20,25,30,31]. These differences may be due to a specific residue associated with the MIDAS, particularly the β3 residue Ala^252^ and corresponding Ala in the β1 integrin compared to the analogous Asp residue in the β2 and β7 integrins. Mutations in the ADMIDAS of α4β7 and αLβ2 integrins increased the binding and adhesion abilities compared to the wild type (WT), while the same mutations in the α2β1, α5β1, αVβ3, and αIIbβ3 integrins demonstrated decreased ligand binding and adhesion [4,6,14,20,21,25,31]. Here, we introduced a mutation in the αIIbβ3 to convert this MIDAS associated Ala^252^ to Asp. By combination of this mutant with mutations of one or two ADMIDAS residues, we studied the effects of this residue on ligand binding and adhesion. Since a variety of computational methods have been widely employed to study the dynamics of biological macromolecules [32,33,34,35,36,37], we performed molecular dynamics (MD) simulations followed by binding free energy estimations. The Molecular Mechanics/Generalized Born Surface Area (MM/GBSA) method was used to understand how mutations at the residue Ala^252^ affect the structure and dynamics of the metal ion binding sites and the binding affinity between αIIbβ3 WT and mutants and the RGD ligand. Our results provided detailed structural and kinetic characterizations for the β3 I domain of αIIbβ3 integrin in the WT and Ala^252^ mutant forms. Our results suggest that the variation in this MIDAS associated residue accounts for the differences in ligand binding and adhesion to fibrinogen among different integrins, and it accounts for the conflicting results of ADMIDAS mutations within different integrins. This study sheds more light on the role of the MIDAS associated residue pertaining to ligand binding and adhesion and suggests that this residue may play a pivotal role in integrin-mediated cell rolling and firm adhesion.

## Materials and Methods

### Plasmid Construction and Expression

Plasmids with sequences for full-length human αIIb and β3 were subcloned into pEF/V5-HisA and pcDNA3.1/Myc-His(+), respectively [26,38]. The β3 mutants A252D, A252D/D126A, A252D/D127A, and A252D/D126A/D127A were made using site-directed mutagenesis with the QuikChange kit (Stratagene, La Jolla, CA). Constructs were transfected into HEK293T cells (American Type Culture Collection, Manassas, VA) using FuGENE transfection kit (Roche Applied Sciences, Indianapolis, IN) according to the manufacturer’s instructions. The expression levels of αIIb and β3 were detected by flow cytometry staining with the following monoclonal antibodies: AP3 (nonfunctional anti-β3 mAb, American Type Culture Collection), 7E3 (anti-β3 mAb), 10E5 (anti-αIIb mAb, kindly provided by B.S. Coller, Rockefeller University, New York, NY), and LM609 (anti-αV mAb).

Two Color Ligand Binding Assay on HEK293T Transfectants- Soluble binding of ligand mimetic IgM PAC-1 (BD Biosciences, San Jose, CA) and Alexa Fluor 488-labeled human fibrinogen (Enzyme Research Laboratories, South Bend, IN) was determined as previously described [38]. Briefly, transfected cells suspended in 20 mM HEPES-buffered saline, pH 7.4 (HBS) supplemented with 5.5 mM glucose and 1% bovine serum albumin (BSA) were incubated on ice for 30 min with 10 µg/mL PAC-1 or 60 µg/mL Alexa Fluor 488-conjugated human fibrinogen in the presence of either 5 mM EDTA, 5 mM Ca2+, or 1 mM Mn2+. For PAC-1 binding, cells were washed and stained with FITC-conjugated anti-mouse IgM on ice for another 30 min before being subjected to flow cytometry. Cells were also stained in parallel with Cy3-conjugated anti-β3 mAb AP3. All samples were properly gated to the correct population to ensure consistent expression. Binding activity is presented as the percentage of the mean fluorescence intensity (MFI) of PAC-1 or fibrinogen staining after background subtraction of the staining in the presence of EDTA, relative to the MFI of the AP3 staining. An unpaired t-test was conducted for each WT and/or mutant comparison using a P-value of 0.05 as statistically significant.

### Cell Adhesion Assay

Cell adhesion on immobilized human fibrinogen was assessed by the measurement of cellular lactate dehydrogenase (LDH) activity as previously described [39]. Briefly, cells suspended in HBS supplemented with 5.5 mM glucose, 1% BSA, and 1 mM Ca^2+^ were added into flat bottom 12-well plates (1 x 10^5^ cells/well) precoated with 20 µg/mL human fibrinogen and blocked with 1% BSA. After incubation at 37°C for 30 min, wells were washed three times with HBS supplemented as indicated above. Remaining adherent cells were lysed with 1% Triton X-100, and LDH activity was assayed using the Cytotoxicity Detection Kit (Roche Applied Science) according to the manufacturer’s instructions. Cell adhesion was expressed as a percentage of bound cells relative to total input cells. An unpaired t-test was conducted for each WT and/or mutant comparison using a P-value of 0.05 as statistically significant.

### Cell Spreading and Microscopy

Glass bottom 6-well plates (MatTek Corporation, Ashland, MA) were coated with 20 µg/mL human fibrinogen in phosphate-buffered saline at pH 7.4 (PBS) overnight at 4°C, and then blocked with 1% BSA at RT for 1 h. The transiently transfected HEK293T cells were detached with trypsin/EDTA, washed three times with DMEM, and seeded on fibrinogen-coated plates. After incubation at 37°C for 1 h, cells were washed three times with PBS and fixed with 3.7% formaldehyde in PBS at RT for 10 min for microscopy.

Differential interference contrast (DIC) imaging was conducted on a Leica TCS SP2 spectral confocal system coupled to a DM IRE2 inverted microscope with a 63X oil objective. For quantification of cell spreading area, outlines of 100 randomly selected adherent cells were generated, and the area (µm^2^) contained within each of these regions was measured using ImageJ software (Bethesda, Maryland). An unpaired t-test was conducted for each WT and/or mutant comparison using a P-value of 0.05 as statistically significant.

### Molecular Dynamics Simulations Design

**Figure 1:**
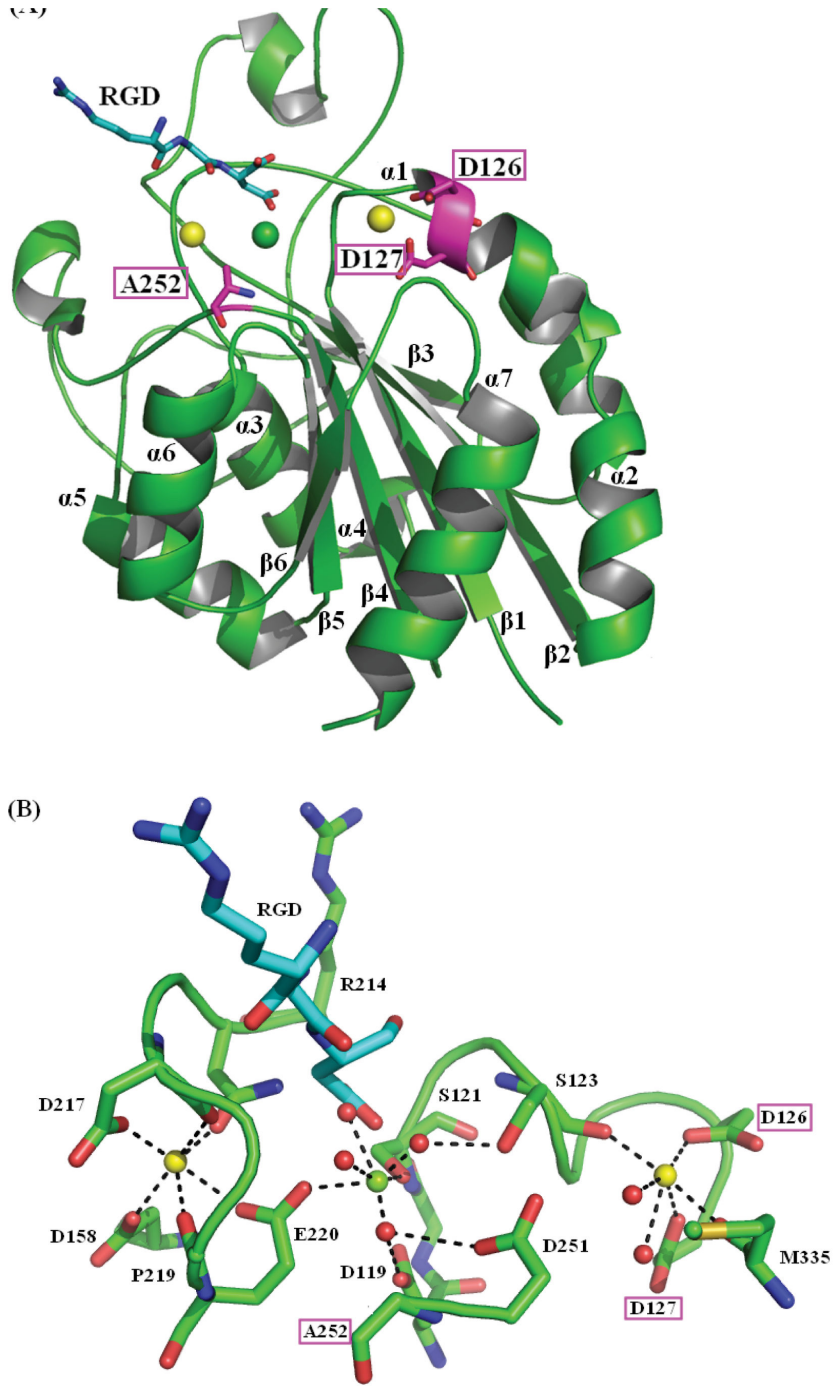
The initial structure of the β I domain of integrin αIIbβ3. Amino acid residues from 109 to 354 of the β I domain of integrin αIIbβ3 (PDD: 3FCS) in Mg^2+^-Ca^2+^-Ca^2+^ state, and the ligand RGD coordinates were modeled by manual. (A) The complex structure of wild-type β I domain/RGD; (B) The local ligand metal-binding sites. The β I domain of integrin αIIbβ3 is shown in cartoon and colored green. The three residues Asp126, Asp127 and Ala252 are shown in stick and colored magenta. Ligand is shown in stick and colored cyan. N and O atoms are colored in blue and red, respectively. Magnesium is a green sphere, calcium ions are yellow, and crystal water molecules are red (Wat692, Wat756, Wat757, Wat761 in MIDAS site and Wat693, Wat694 in ADMIDAS site). Polar coordination between O atoms and metal ions are shown by dashed black lines. Figures are produced by PyMOL (www.pymol.org).

The X-ray crystallographic structure of wild-type (WT) αIIbβ3 integrin was obtained from the Protein Data Bank (PDBID code: 3FCS) [17]. As a basis of comparison, a typical model of the β3 I domain, including isolated amino acid residues from 109 to 354, the MIDAS Mg^2+^, the ADMIDAS and SyMBS Ca^2+^ ions, and the ligand RGD, was formed using published structure (PDBID code: 2DVR) by manual [40], which is denoted as Mg^2+^-Ca^2+^-Ca^2+^ (Figure 1). All water molecules in the crystal structure of the β3 I domain were kept in the starting model. The initial model of the complex formed by Mn^2+^ was constructed, based on the aforementioned Mg^2+^-Ca^2+^-Ca^2+^ model, by replacing Mg^2+^ or Ca^2+^ with Mn^2+^ in all three sites (Mn^2+^-Mn^2+^-Mn^2+^). The selected Ala^252^ residue was mutated to Asp and combined with mutations of one or two ADMIDAS residues Asp^126^ and/or Asp^127^ to Ala using the Mutate Monomers component of the SYBYL 8.1 software from Tripos. Ten molecular systems were constructed for WT and A252D mutants in the Mg^2+^-Ca^2+^-Ca^2+^ and Mn^2+^-Mn^2+^-Mn^2+^ states, respectively.

### Molecular Dynamics Simulations

The parallel version of AMBER 10 package was used to prepare the protein and ligand parameters and conduct MD simulations employing AMBER ff03 all-atom force fields [41,42]. The force field parameters for cations Mn^2+^ and Ca^2+^ were obtained from the AMBER contributed parameters database at http://www.pharmacy.manchester.ac.uk/bryce/amber [43]. Hydrogen atoms were added to the amino acids. Using the TLEAP program, all MD simulations were carried out by applying periodic boundary conditions in an explicit water box of TIP3P water molecules with a margin of at least 12 Å from any edge of the box to any atom of the solute molecules [44]. The Cl^-^ or Na^+^ counterions were added to neutralize the solvated systems. The solvated systems were minimized using SANDER module to eliminate of any unfavorable contacts, while the temperature was gradually heated from 0 K to 300 K in 100 ps then equilibrated for 400 ps with a weak restraint at 300 K. Finally, all restraints were removed, and 30 ns production MD simulations were run for each system at a constant temperature of 300 K and a constant pressure of 1 atm. A cutoff radius of 10 Å for both non-bonded electrostatic and van der Waals interactions were used in all simulations. Bond lengths involving hydrogens were restrained by the SHAKE algorithm [45]. The long-range electrostatic forces were treated using the particle mesh Ewald (PME) method [46]. A time step was set to 2 fs.

### Structural Analysis

The trajectories were recorded at every 1 ps and were analyzed using PTRAJ module of the AMBER 10 program. The root-mean-square deviations (RMSD) of the backbone atoms were monitored to evaluate the equilibration of the system. Other analyses were also made concerning the estimation of the backbone atomic root-mean-square fluctuations (RMSF), principal component analysis (PCA), and hydrogen bond interactions. The Molecular Mechanics/Generalized Born Surface Area (MM/GBSA) method was performed to estimate the binding free energy for ligand RGD and integrin complexes. The interactions between RGD and each residue in the β3 I domains were calculated using the MM/GBSA decomposition program implemented in AMBER10.0. All energy components were calculated using 200 snapshots from the last 10 ns of MD simulations.

## Results

### Design of Mutant αIIbβ3 Integrins

While studying the variation in crystal structures between the liganded and unliganded αIIbβ3 [16,17] and the unliganded αXβ2 [47], we proposed that the β3 Ala^252^ might contribute significantly to the difference in ligand binding and adhesion among different integrins [25,31]. While this Ala^252^ residue does not appear to interact with MIDAS Mg^2+^ ion, the corresponding residue Asp^243^ in the αXβ2 integrin [47] does appear to contribute negative charge to the MIDAS that could have an effect on the ligand binding ability. Therefore, we made the β3 mutant A252D, which was expected to affect the negativity of the β3 MIDAS resulting in decreased ligand binding affinity. Furthermore, we made three αIIbβ3 mutants in which we paired A252D with mutations of one or two ADMIDAS residues. By replacing the Asp of the ADMIDAS residues with the Ala, the function of the ADMIDAS was expected to be affected, as previously described [25,31].

### Expression of Mutant αIIbβ3 Integrins

**Figure 2:**
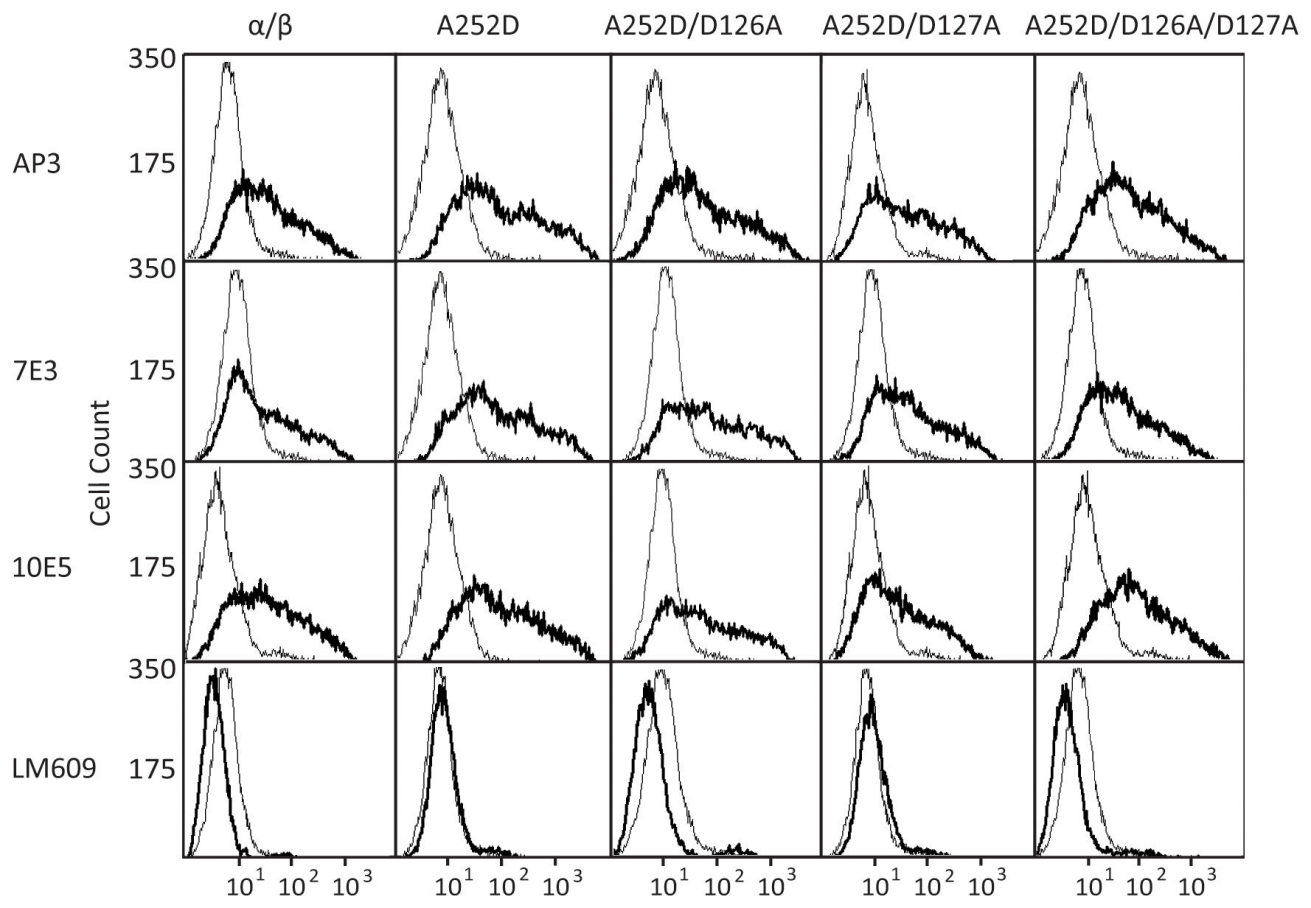
Expression of WT and Mutant αIIbβ3 Integrins. Immunofluorescent flow cytometry. HEK293T transfectants were labeled with AP3 (anti-β3), 7E3 (anti-β3), 10E5 (anti-αIIb), and LM609 (anti-αV). Thick and thin lines show labeling of the αIIbβ3 transfectant and the mock transfectant, respectively.

To determine the expression of integrins on the cell surface, the WT and αIIbβ3 mutants were transfected into HEK293T cells and subjected to immunostaining flow cytometry. Four monoclonal antibodies were used to detect integrin folding and expression: AP3 recognizes the β3 hybrid domain, 7E3 binds to the β3 I domain, 10E5 recognizes the αIIb β-propeller domain, and LM609 binds to the αV subunit. When the WT and mutant αIIbβ3 integrins were cotransfected, LM609 did not bind to the cells (Figure 2), confirming that no endogenous αV was expressed that could potentially complicate our experiments. WT and mutant integrins bound AP3, 7E3, and 10E5 with similar levels (Figure 2), suggesting that all mutations seemed to have little effect on integrin folding and expression on the cell surface.

### Regulation of PAC-1 and Fibrinogen Binding by the MIDAS Associated Residue

**Figure 3:**
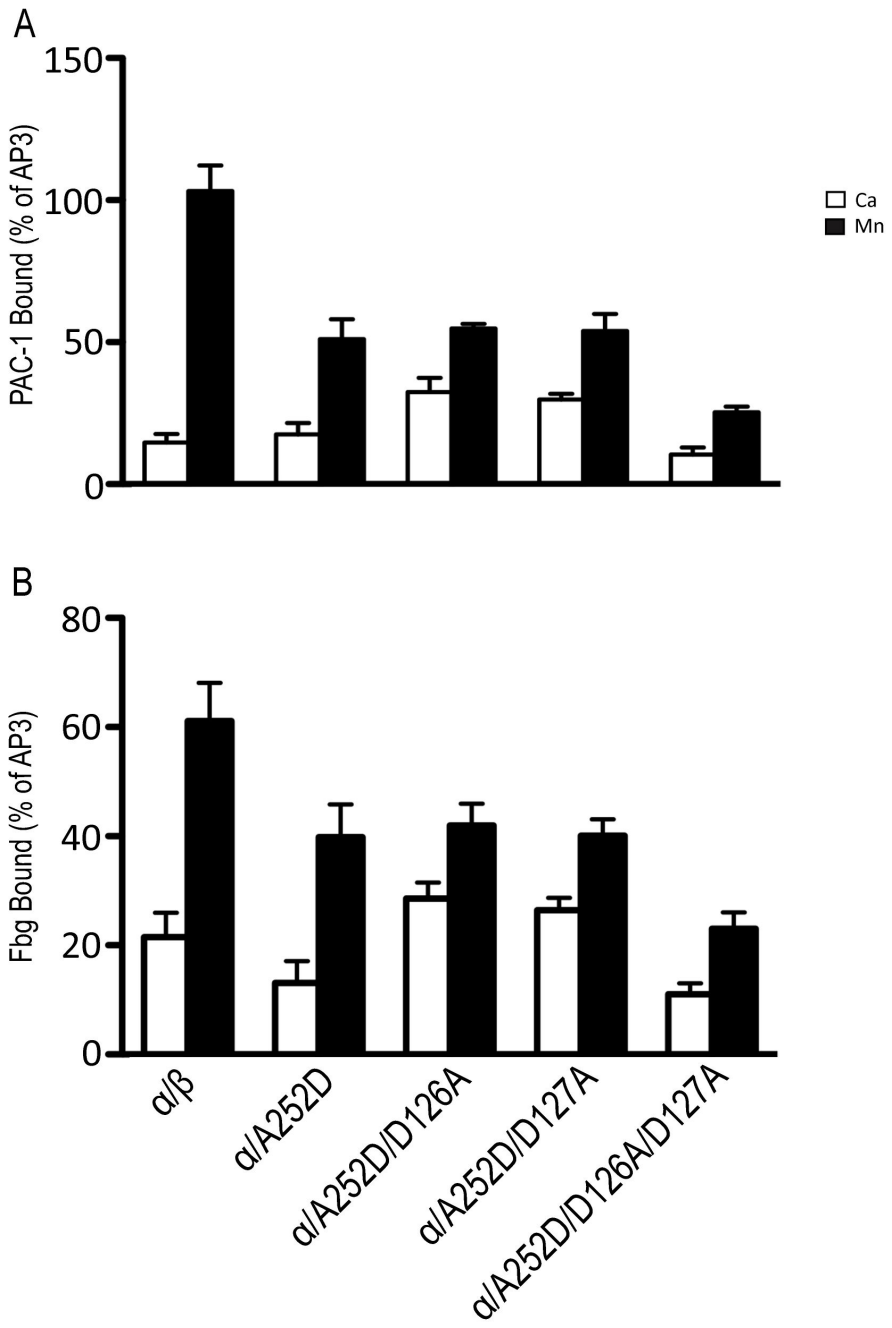
Soluble Ligand Binding with PAC-1 and Fibrinogen. Cells were incubated with (A) PAC-1 in the presence of 5 mM Ca^2+^ or 1 mM Mn^2+^ or (B) FITC-fibrinogen in the presence of 5 mM Ca^2+^ or 1 mM Mn^2+^ as indicated. Binding activities were determined by flow cytometry and expressed as described in Materials and Methods. Error bars are standard deviation (SD). An unpaired t-test with n=1000 for each group was conducted and showed to be statistically significant with a p-value <0.05 between WT and A252D for PAC-1 binding in Mn^2+^ conditions and fibrinogen binding in both Ca^2+^ and Mn^2+^ conditions, between WT and A252D/D126A as well as WT and A252D/D127A for PAC-1 and fibrinogen binding in both Ca^2+^ and Mn^2+^ conditions, and between A252D and A252D/D126A as well as A252D and A252D/D127A for PAC-1 and fibrinogen binding in Ca^2+^ conditions. However, insignificant results with a p-value >0.05 occurred between WT and A252D for PAC-1 binding in Ca^2+^ conditions and between A252D and A252D/D126A as well as A252D and A252D/D127A for PAC-1 and fibrinogen binding in Mn^2+^ conditions.

Two-color flow cytometry was used to determine the fibrinogen and ligand mimetic antibody PAC-1 binding of the WT and mutants. In normal physiological Ca^2+^ conditions, WT αIIbβ3 bound very little ligand-mimetic PAC-1 antibody or fibrinogen, which is consistent with previous reports [25,48]. In the presence of Mn^2+^, the WT bound PAC-1 and fibrinogen with higher affinity (Figure 3). The MIDAS associated mutant A252D showed statistically significant reduced ligand binding affinity to fibrinogen compared to the WT in Ca^2+^, while both seemed to have similar low affinity to PAC-1. In the presence of Mn^2+^, the A252D mutant showed an increase in PAC-1 and fibrinogen binding, but was significantly lower than the WT, suggesting that the increased negative charge from the Asp reduced the ligand binding ability of the αIIbβ3 MIDAS (Figure 3).

When paired with mutations of one ADMIDAS residue, the double mutants A252D/D126A and A252D/D127A showed slightly increased, but statistically significant, binding to fibrinogen in Ca^2+^ conditions compared to the WT. In Mn^2+^ conditions, these double mutants showed increased fibrinogen binding compared to the Ca^2+^ conditions but lower binding than the WT. Similar results were obtained for PAC-1 binding, indicating that the double mutants have higher affinity compared to the WT in Ca^2+^ conditions. Interestingly, these double mutants showed an increased binding to PAC-1 and fibrinogen in the Ca^2+^ conditions compared to the A252D mutant alone, while both the A252D and the double mutants bound PAC-1 and fibrinogen with relatively similar affinities in Mn^2+^ conditions (Figure 3). These results suggest that mutation of one ADMIDAS residue is able to compensate for the ligand-binding defect of the A252D mutant in the Ca^2+^ conditions.

By contrast, when the A252D mutant was combined with mutation of two ADMIDAS residues, the A252D/D126A/D127A mutant showed almost no binding to PAC-1 or fibrinogen in Ca^2+^ conditions, and Mn^2+^ could only slightly increase the ligand binding of this mutant, indicating that the triple mutant abolished the ADMIDAS coordination with the Ca^2+^ ion that caused almost no binding to either ligand (Figure 3).

### Effects of the A252D Mutant on Adhesion and Spreading

**Figure 4:**
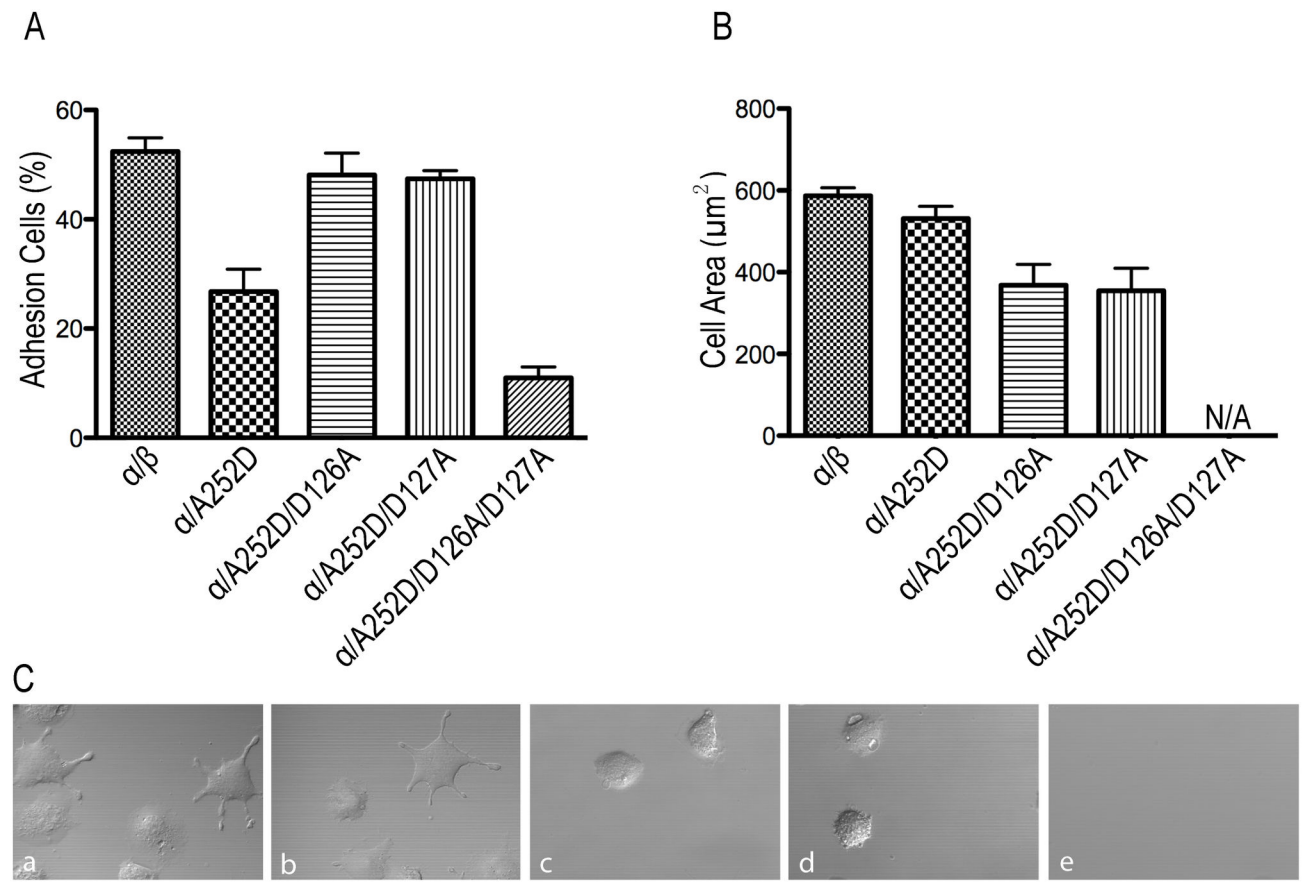
Cell adhesion and spreading. A. Adhesion of HEK293T transfectants to surfaces coated with 20 µg/mL fibrinogen. The amount of bound cells was determined by measuring LDH activity as described in Materials and Methods. Data are representative of three independent experiments, each in triplicate. Error bars are SD. B. Quantification of the areas of adhering/spreading cells as described in Materials and Methods. C. DIC images of HEK293T transfectants after adhering to immobilized fibrinogen at 37°C. a: WT; b: A252D; c: A252D/D126A; d: A252D/D127A; e: A252D/D126A/D127A. The images are representatives of three independent experiments.

Using cytotoxicity detection of LDH, the percentage of adherent cells to immobilized fibrinogen was calculated by ratio of adherent cells to total input cells. Over half of the WT cells adhered to immobilized fibrinogen, while only one fourth of the A252D mutant cells adhered, which correlated to its reduced ligand binding affinity (Figure 4A). The double mutants A252D/D126A and A252D/D127A adhered to the immobilized fibrinogen at similar levels as the WT. The triple mutant A252D/D126A/D127A nearly abolished adhesion to fibrinogen, which relates to its relatively low ligand binding affinity (Figure 4A).

By studying cell spreading, the role of the MIDAS associated residue on integrin outside-in signaling could be determined by coating HEK293T transient transfectants on immobilized fibrinogen at 37°C for 1 h, followed by fixation and microscopic analysis. WT transfected cells showed normal cell adhesion and spreading (Figure 4B). Although the A252D mutant showed reduced ligand binding affinity and adhesion compared to the WT, the adherent cells underwent outside-in signaling and spread similarly to the WT (Figure 4C). Intriguingly, A252D/D126A and A252D/D127A were able to adhere, but showed defective cell spreading compared to the WT (Figure 4C). The exact mechanism of the impaired spreading/outside-in signaling remains elusive due to the complexity and ambiguity of the mutant structures. Finally, no spreading data were obtained for the A252D/D126A/D127A mutant, since there was almost no adhesion (Figure 4B).

### Equilibration of WT and Mutant αIIbβ3 Integrin β3 I Domain Systems

The present study includes a total of 300ns all-atom, explicit solvent MD simulations for WT and mutant αIIbβ3 integrin β I domains. To explore the degree of similarity of snapshots of MD simulations with the reference structures of the WT and mutant β3 I domains, RMSD values of all backbone atoms were calculated using their initial structures as references (Figure S1). The results revealed that all the systems increased initially and then leveled off at ~1.5 Å, indicating the equilibrium had been reached after 20 ns of MD simulations and all trajectories of those complexes were quite stable and could be used for further analysis.

Meanwhile, the RMSF (average RMSD of each residue) values were also calculated to evaluate regions exhibiting important dynamic behavior (Figure S2). Under the Mg^2+^-Ca^2+^-Ca^2+^ condition, the single mutant A252D was more flexible than WT, especially in some regions, such as the β2-β3, β4-α5, α6-β6, β6-α7 loops, and α1, α4, α5, α6, α7 helices; while the other three mutants showed more stability than the WT in these regions. In order to describe the explicit picture of the global movements and reveal relevant biological functions effectively, the dominant low-frequency motions were characterized by PCA. In this way, the large-scale motions were extracted from each complicated MD trajectory in order to present the internal collective motions of the protein. It is well known that the first few principal components (PCs) account for most of the variance in the observed motion, highlighting the major conformations sampled in a MD simulation trajectory. The first component (PC1) corresponds to the largest contribution, the second largest component (PC2) represents the next, and so on [49].

**Figure 5:**
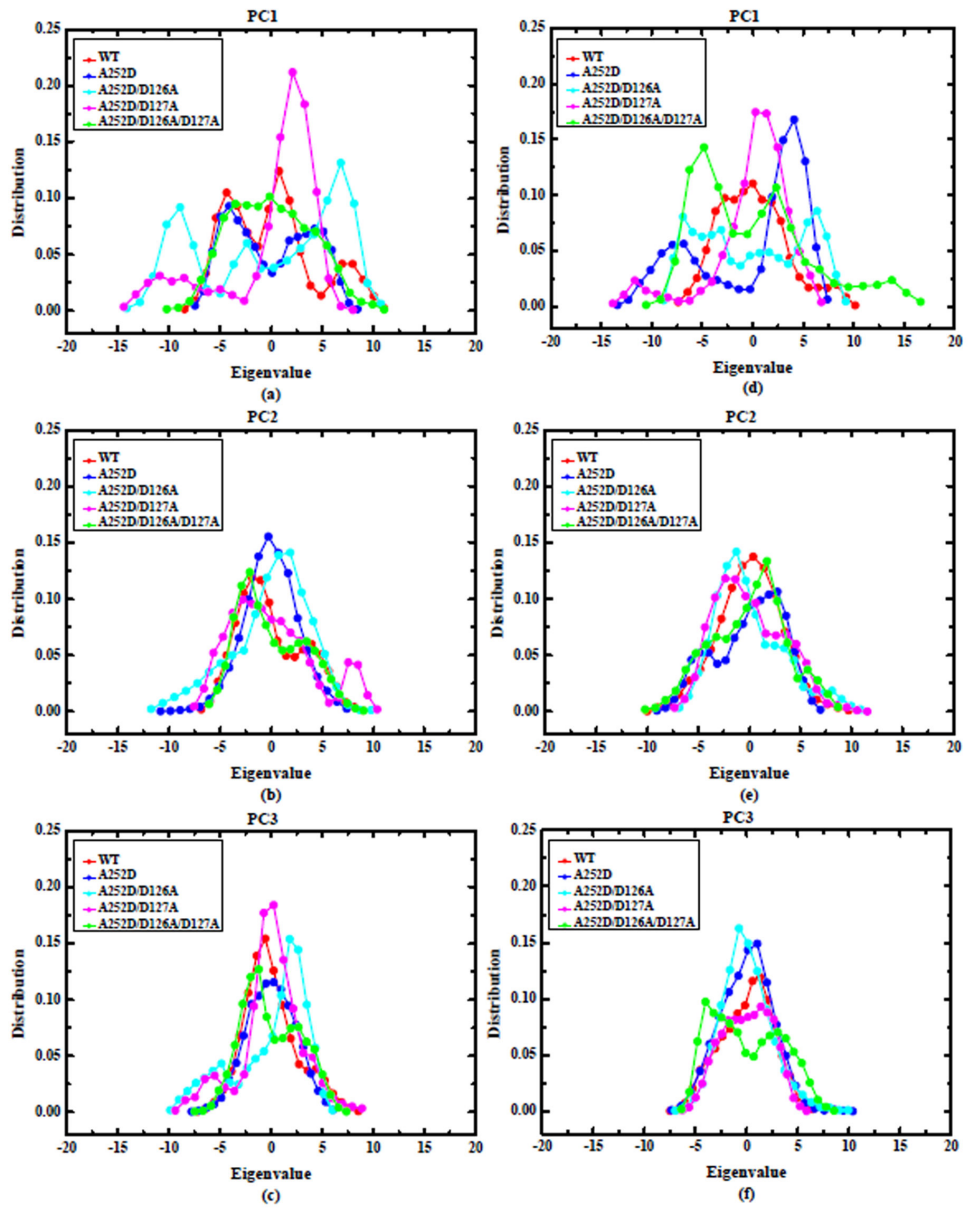
The distributions of the eigenvalues on the first, second, and third principal components (PC). The distributions of the eigenvalues on the PC1, PC2, and PC3 are demonstrated for Mg^2+^-Ca^2+^-Ca^2+^ [(a), (b), and (c)] and Mn^2+^-Mn^2+^-Mn^2+^ [(d), (e), and (f)] states.

The eigenvalue involved in each vector describes the variance of the molecular motion along that vector. The projections of the first three PCs, which serve to indicate the conformational preferences of the WT and mutant β3 I domain, and the distribution of the eigenvalues for each system are shown in Figure 5. Almost all of the probability distributions display multimodality, indicating that the ten simulated systems have to overcome some energy barriers along these PCs. It can be seen in the eigenvalue distributions that mutations could introduce large differences in the movement along the first three PCs. The mutant A252/D126A and A252D/D127A were more flexible than other samples in the Mg^2+^-Ca^2+^-Ca^2+^ state. These two mutants had the widest multimodal probability distributions along the first three PCs among all distributions. On the contrary, the A252D and A252D/D126A/D127A exhibited more flexibility than others in the Mn^2+^-Mn^2+^-Mn^2+^ state. In both states, a high peak appeared in the mutant A252D/D127A along the PC1 (Figure 5A & D). These results confirmed that the mutations could affect the dynamic behavior of the β3 I domain, with varying effects between the Mg^2+^-Ca^2+^-Ca^2+^ and Mn^2+^-Mn^2+^-Mn^2+^ states.

### MM/GBSA Calculation and Decomposition of Binding Free Energies

To express the strength of ligand RGD affinity towards the β3 I domain of αIIbβ3 integrin in the WT and mutant forms, 200 snapshots were taken from the last 10 ns of MD trajectories for analysis of the binding free energy using MM/GBSA method. The configurational entropy was not considered in this approach. It is generally assumed that the entropy changes were similar when involving one ligand binding to a set of different proteins. The binding free energy in the solvent environment can be expressed as

ΔG_bind_ =ΔE_ele_ + ΔE_vdw_ +ΔG_np_ +ΔG_ele_

where, ΔE_ele_: electrostatic energy in the gas phase

ΔE_vdw_: van der Waals energy
ΔG_np_: non-polar solvation energy
ΔG_ele_: polar solvation energy

Table 1 lists the components of molecular mechanics (ΔE_ele_ and ΔE_vdw_) and solvation energies (ΔG_np_ and ΔG_ele_) for WT and mutant systems of both states. According to energy analysis, ΔE_ele_ in the gas phase provided the major favorable contribution to ligand binding, whereas ΔG_ele_ tremendously impaired binding. The ΔE_vdw_ and ΔG_np_ contributed slightly favorably to the ligand binding. For the Mg^2+^-Ca^2+^-Ca^2+^ state, the mutations resulted in slight changes in ΔE_vdw_ and almost no effect on ΔG_np_, with an increase in both ΔE_ele_ and ΔG_ele_ except for the A252D mutant that resulted in an increased negative MIDAS for ligand binding. For the Mn^2+^-Mn^2+^-Mn^2+^ state, the mutations led to slight change in ΔE_vdw_, almost no effect on ΔG_np_, and decrease in both ΔE_ele_ and ΔG_ele_ except for the triple mutant.

**Table 1:** Binding free energies computed by the MM/GBSA method (neglecting the configurational entropy, kcal/mol).

	**Complex**	**ΔE_ele_**	**ΔE_vdw_**	**ΔG_np_**	**ΔG_ele_**	**ΔG_solv_**	**ΔE_ele_+ΔG_ele_**	**ΔG_bind_**
**(Mg^2+^-Ca^2+^-Ca^2+^)**	WT	-243.50±20.92	-11.66±4.21	-3.35±0.31	235.83±18.16	232.48±18.00	-7.67±5.85	-22.68±4.54
	A252D	-225.69±35.95	-11.77±3.90	-3.12±0.51	222.78±36.47	219.66±36.03	-2.91±5.13	-17.80±3.66
	A252D/D126A	-302.70±27.81	-12.57±3.57	-3.64±0.17	290.97±23.39	287.33±23.29	-11.73±6.55	-27.94±5.38
	A252D/D127A	-303.69±40.41	-10.22±4.97	-3.66±0.37	290.94±33.29	287.28±33.08	-12.75±9.94	-26.63±7.26
	A252D/D126A/D127A	-377.10±58.18	-8.94±4.01	-3.73±0.15	368.66±58.13	364.93±58.06	-8.43±5.19	-21.10±3.59
**(Mn^2+^-Mn^2+^-Mn^2+^)**	WT	-343.63±21.05	-12.58±4.35	-4.68±0.37	342.66±21.49	337.98±21.27	-0.97±5.89	-18.24±4.02
	A252D	-272.53±24.35	-12.14±3.65	-3.64±0.24	281.37±25.14	277.73±24.98	8.84±6.46	-6.95±5.81
	A252D/D126A	-155.59±16.45	-14.62±3.24	-2.80±0.24	159.69±14.10	156.89±14.15	4.10±5.48	-13.32±5.06
	A252D/D127A	-280.63±23.01	-12.67±4.42	-3.72±0.25	285.12±18.34	281.40±18.28	4.49±7.23	-11.90±6.18
	A252D/D126A/D127A	-415.50±29.51	-1.21±5.52	-3.43±0.48	417.00±25.69	413.57±25.54	1.50±6.74	-0.72±5.96

From Table 1, the two double mutants A252D/D126A and A252D/D127A showed stronger binding free energies which resulted in an increased binding efficacy, while the single mutant A252D and the triple mutant A252D/D126A/D127A showed weaker affinities compared to WT in Mg^2+^-Ca^2+^-Ca^2+^ state. Similarly, the ligand binding affinity sharply decreased in the A252D mutant, increased in the double mutants A252D/D126A and A252D/D127A, and nearly abolished in the triple mutant A252D/D126A/D127A in the Mn^2+^-Mn^2+^-Mn^2+^ state. The trends of these calculated binding free energies were in excellent accordance with the mutagenesis experimental results, providing further evidence that the MIDAS associated residue affects the integrin ligand binding affinity.

To acquire insight into the contributions of each residue to binding free energies and the understanding of binding mechanisms with residue mutations in different divalent cation states, binding free energies were decomposed into ligand-residue pairs by using the MM/GBSA method (Table S1, Figures S3 & S4). On the basis of the ligand-protein interaction spectrums, the favorable residues mainly located in the MIDAS and SyMBS regions had the largest contributions to the binding energy, indicating that these residues are crucial for ligand RGD binding. The residue β3 Glu^220^ seemed unfavorable for the ligand-protein interaction. A majority of these important residues are electrostatic. These charged residues and ions in β3 I domain formed strong electrostatic interactions with the side chain of the ligand RDG Asp and Arg.

In Mg^2+^-Ca^2+^-Ca^2+^ state, compared to the WT, the contributions of Asp^251^ in the single mutant A252D system weakened, possibly due to the loss of strong hydrogen bonds formed between the side chain of Asp^251^ and the Arg residue on the ligand. As for the mutant A252D/D126A, the Asp^217^, Ala^218^ and Asp^252^ were more important, partly for the increased occupancies of hydrogen bonds between the ligand Arg and both of the backbone Ala^218^ and the side chain of Asp^252^. The residues Ser^121^, Tyr^122^ and Ser^123^ located in β1-α1 loop, and especially Arg^214^ and Asn^215^ in α2-α3 loop, which formed stable hydrogen bonds with the ligand Asp, were favor in ligand binding in the A252D/D127A mutant. Intriguingly, the Asp^252^ formed stable hydrogen bonds with the ligand Asp, whereas the repulsive interaction energy of ADMIDAS ion was increased. Thus, the total interaction energy was decreased in A252D/D126A/D127A.

In the Mn^2+^-Mn^2+^-Mn^2+^ state, the contribution of ligand Asp in simulated systems was smaller than that of in Mg^2+^-Ca^2+^-Ca^2+^ state, except in the A252D/D126A/D127A mutant, in which this contribution was increased. The contributions of Asp^251^ in the A252D mutant were decreased, which might be the result of losing the strong hydrogen bond between the side chain of Asp^251^ and the ligand Arg. Because of the increased occupancies of hydrogen bonds between the side chain of residues and the ligand Arg, the Ser^121^, Tyr^122^ and Asn^215^ were important in mutant A252D/D126A, while Ala^218^ and Asp^252^ were crucial in the mutant A252D/D127A. Likewise, the increased repulsive interaction energy of ADMIDAS and MIDAS ion resulted in the decreased total interaction energy in A252D/D126A/D127A.

Based on the above analysis of the results, electrostatic interactions played an important role in β3 I domain and ligand RGD interaction, and the tendencies of calculated free binding energies were in excellent accordance with the mutagenesis experimental results of αIIbβ3 integrin. The mutation of residue Ala^252^ to Asp associated with the MIDAS in β3 integrin increased the negative charge of the ligand binding site, resulting in changes in structure, electrostatic interaction, hydrogen bonding, and ultimately the ligand RGD binding affinity. These results suggest that mutation of one ADMIDAS residue was able to compensate for the decreased ligand binding affinity of the A252D mutant, while mutations of two ADMIDAS residues nearly abolished ligand binding.

### The Effects of Residue Mutants on the Dynamic Structures of the β3 I Domain

**Figure 6:**
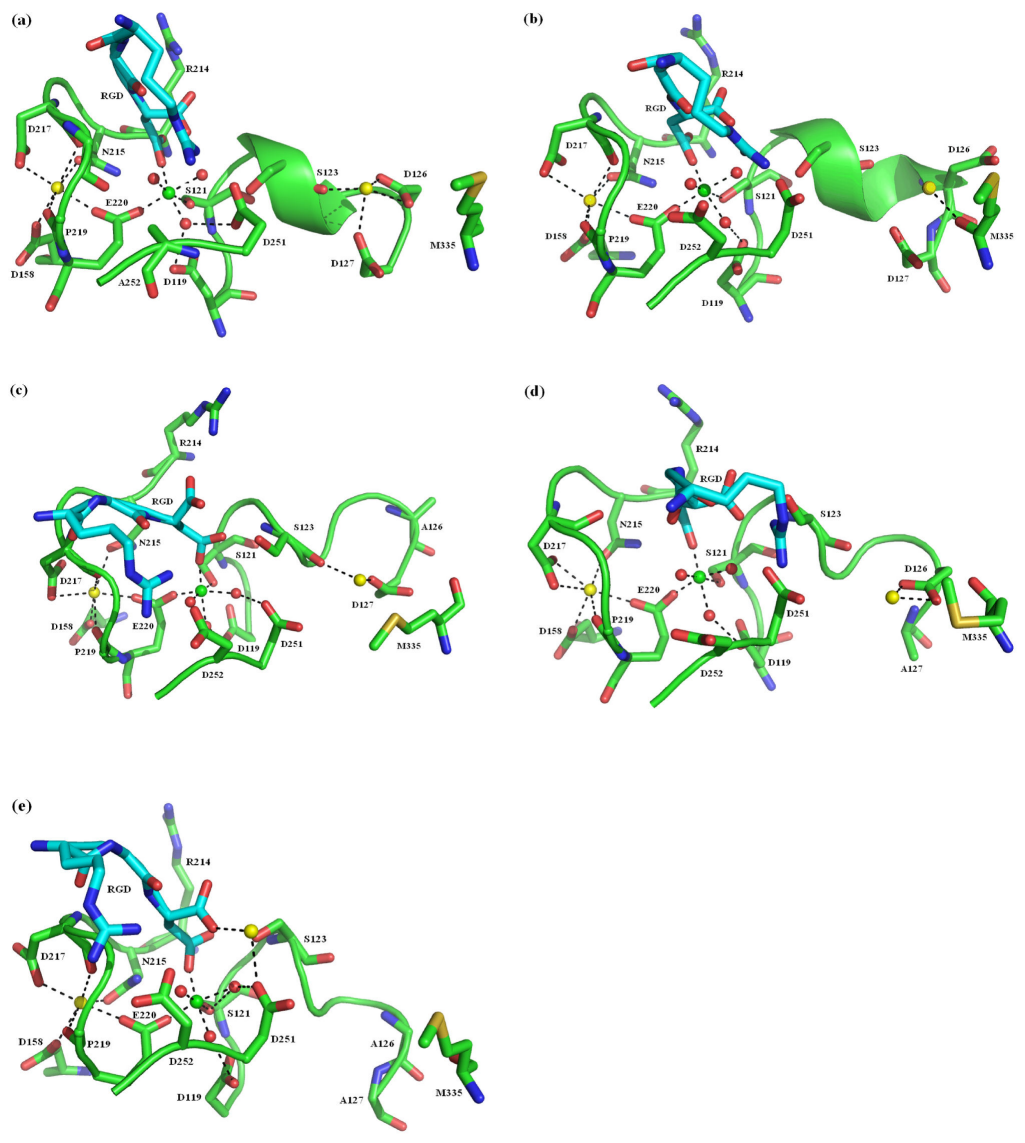
The view of the RGD binding site. Final snapshots of the RGD binding site in (a) wild-type (WT) and mutant (b) A252D, (c) A252D/D126A, (d) A252D/D127A, (e) A252D/D126A/D127A from the molecular dynamics simulations in Mg^2+^-Ca^2+^-Ca^2+^ state. Ligand is shown in stick and colored cyan. N and O atoms involved in metal coordinating are colored in blue and red, respectively. Mg^2+^ is green, Ca^2+^ ions are yellow, and coordinating water molecules are red. Polar coordination between O atoms and metal ions are shown by dashed black lines.

Final snapshots from MD simulations were collected in the WT and mutant simulations in the Mg^2+^-Ca^2+^-Ca^2+^ state. The location of the divalent cations in these snapshots illustrated the various binding modes for ligand-protein interactions and the changes of local structures in different simulations (Figure 6). Several distances (Figures S5 & S6) between divalent cations, the key residues, as well as water molecules were tracked during the whole MD simulations.

The orientation of the MIDAS ion (Figure 6 & S5a-l) was similar in the WT and mutants. Due to the synergy between the MIDAS and SyMBS, the SyMBS Ca^2+^ ion maintained its proper orientation and shared the carboxylic oxygen of Glu^220^, hence stabilizing the MIDAS Mg^2+^ ion during simulations (Figures 6 & S5m-t,y). However, the orientation of the ADMIDAS Ca^2+^ ion (Figures 6 & S5u-x,z) seemed to be much different from the initial structure since the water molecules as well as the residue Met^335^ (except A252D mutant) were all moved farther away. When bound to the ligand RGD, the ADMIDAS Ca^2+^ ion in the WT coordinated the carbonyl oxygen O of Ser^123^, the carboxylic oxygen of Asp^126^, and the Asp^127^ in β1-α1 loop, which both the Asp^126^ and Asp^127^ joined the α1 helix. However, when bound to the ligand RGD, the ADMIDAS Ca^2+^ ion in the A252D mutant coordinated with the backbone carbonyl oxygen O of Met^335^ in β6-α7 loop, stabilizing the β3 integrin in the closed conformation. In addition to the increased negativity of the MIDAS, stabilizing the closed conformation further impaired the ligand binding affinity. Next, the orientation of the ADMIDAS Ca^2+^ ion was partly destroyed in the double mutants. In the A252D/D126A mutant, only Ser^123^ and Asp^127^ coordinated with this cation, while Asp^126^ coordinated with the ADMIDAS ion in the A252D/D127A mutant. Importantly, the Asp^251^ exhibited some drift towards the ADMIDAS and resulted in a more positive MIDAS that possibly increases ligand binding in the double mutants compared to the A252D mutant alone. Finally, as shown in Figures 6E and S5z, the ADMIDAS Ca^2+^ ion moved towards the MIDAS in the A252D/D126A/D127A triple mutant, resulting in structural perturbations including the movement of β1-α1 loop, broken coordination of the Met^335^, and new direct coordination formed between the carboxylic oxygen of both residues (Asp^251^ and ligand Asp) and this metal ion, respectively. The ADMIDAS function in this mutant was lost due to the complete loss of coordination with the ADMIDAS Ca^2+^ ion and the repulsive interaction energies between ADMIDAS residues and the ligand increased, therefore, the ligand binding affinity was reduced. In each simulated system, the α1 helix moved inward and the β6-α7 loop moved downward relative to its position in the initial crystal structure.

In summary, compared to the crystal structure, both orientations of the MIDAS Mg^2+^ ion and SyMBS Ca^2+^ ion were very stable and the distance between the two ions remained unchanged during simulations (Figure S5y). However, the ADMIDAS Ca^2+^ ion caused a large disturbance in the conformation of the β1-α1 loop, especially in the mutant complexes. The two coordinated crystal water molecules were all moved farther away, and the coordination with Asp^126^, Asp^127^, and/or Met^335^ was broken in these different systems.

## Discussion

In this study, we showed that mutating the β3 MIDAS associated residue Ala^252^ to Asp reduced the ligand binding and adhesion ability but maintained normal outside-in signaling. In combination with a mutation of one ADMIDAS residue from Asp to Ala, the double mutants showed increased binding to ligands but impaired outside-in signaling compared to the A252D mutant alone. But if two ADMIDAS residues were mutated, the mutant almost completely abolished ligand binding. These results echo similar results of the αVβ3 that suggests variation in this residue among different integrins causes differences in ligand binding and adhesion. We have also used MD to simulate these mutations in the β3 I domain MIDAS and ADMIDAS and to determine the effects of the MIDAS associated residue on ligand binding affinity in the αIIbβ3 integrin. Data from the MD simulations support the results of the mutagenesis studies regarding the effects of the MIDAS associated mutant on ligand binding and adhesion.

Comparing the crystal structures of the unliganded [17] and liganded [16] αIIbβ3, the non-polar β3 MIDAS associated residue Ala^252^ does not associate with the MIDAS metal ion, thus not participating in the process of ligand binding. When the αIIbβ3 integrin undergoes inside-out activation and changes conformation from the closed headpiece [17] low affinity state to the open headpiece [16] high affinity state, the Asp^251^ shifts from coordinating with the MIDAS metal ion to the ADMIDAS Ca^2+^ ion, while the Ala^252^ also shifts away from the MIDAS but does not coordinate with the ADMIDAS ion. By shifting the Asp^251^ away from the MIDAS towards the ADMIDAS, the MIDAS becomes more positively charged able to bind ligands with higher affinity [31]. Previous studies have shown that when one of the ADMIDAS residues is mutated, the resulting mutant can either increase or decrease the integrin for ligand binding [4,6,14,20,21,25,31]. Our current work suggests that the MIDAS associated residue (Ala in β1 and β3, and Asp in β2 and β7) accounts for these discrepancies. When one of the α5β1, α2β1, αVβ3, or αIIbβ3 ADMIDAS residues was mutated from Asp to Ala, ligand binding affinity decreased compared to the WT because the loss of coordination with the ADMIDAS Ca^2+^ ion abolished ADMIDAS function [14,20,25,31]. However, mutations in one of the ADMIDAS residues from Asp to Ala in the α4β7 and αLβ2 integrins [4,6,21] resulted in increased ligand binding affinity compared to the WT possibly due to the MIDAS associated residue Asp shifting toward the ADMIDAS in the open headpiece conformation and retaining the ADMIDAS Ca^2+^ ion and thus its function. This shift would also allow the MIDAS to become even more positive and bind ligands with higher affinity than the WT.

We chose to determine if this MIDAS associated residue Ala^252^ in the αIIbβ3 was changed to Asp would yield similar ligand binding and adhesion results to the αLβ2 and α4β7 integrins, as previously conducted in the αVβ3 integrin [31]. Mutation of the β3 Ala^252^ to Asp resulted in lower ligand binding affinity and immobilized ligand adhesion ability. Interestingly, when combined with a mutation in one of the αIIbβ3 ADMIDAS residues, affinity for ligands and adhesion ability increased. Nevertheless, when both ADMIDAS residues were mutated and combined with the A252D mutation, ADMIDAS function was lost due to the loss of coordination with the ADMIDAS Ca^2+^ ion resulting in decreased ligand binding affinity and adhesion. These mutagenesis results, along with results from the αVβ3, support our hypothesis that the variation in the MIDAS associated residue results in differences in ligand binding and adhesion. MD simulations further strengthen this hypothesis by demonstrating that variations in the MIDAS associated residue account for differences in binding properties and structural integrity. In either the Mg^2+^-Ca^2+^-Ca^2+^ or Mn^2+^-Mn^2+^-Mn^2+^ states, the A252D mutant showed decreased binding free energy compared to the WT. When combined with a mutation in one ADMIDAS residue, the binding free energy increased compared to the A252D mutant alone, while the triple mutant A252D/D126A/D127A showed very low binding free energy.

The dynamic structure of the β3 I domain in the mutant structures revealed that both orientation of the MIDAS and SyMBS ions remained the same while the ADMIDAS ion was different compared to the WT. Due to the synergy between MIDAS and SyMBS, the SyMBS ion maintained its proper orientation and shared the carboxylic oxygen of Glu220, hence stabilizing the MIDAS ion occupancy. In addition, water molecules also played an important role in maintaining the coordination of MIDAS ion. On the contrary, the ADMIDAS ion showed a large disturbance in the conformation of the β1-α1 and β6-α7 loops and broken coordination of Met^335^ compared to the WT, providing support that the Asp^251^ along with the Asp^252^ shift toward the ADMIDAS and coordinate with the Ca^2+^ ion to stabilize and maintain ADMIDAS function in the liganded state. First, the A252D mutation increased the negativity of MIDAS and thus was not in favor of ligand binding. The coordination of Met^335^ and ADMIDAS Ca^2+^ ion in A252D mutant inhibited the conformational change and stabilized β3 integrin in the closed conformation in the Mg^2+^-Ca^2+^-Ca^2+^ state, further decreasing the ligand binding affinity of this mutant. Nevertheless, in the double mutants, the Asp^251^ drifted toward the ADMIDAS to coordinate with the Ca^2+^ ion and resulted in a more positive MIDAS for ligand binding. Finally, due to the complete loss of coordination with the ADMIDAS Ca^2+^ ion, the repulsive interaction energies between the ADMIDAS residues and ligand were increased in the triple mutant, resulting in the decreased total interaction energy. The results of these mutagenesis studies and MD simulations provide evidence that the MIDAS associated residue accounts for the variation in ligand binding and adhesion to fibrinogen among integrins.

It is interesting that both β2 and β7 integrins mediate cell rolling and firm adhesion, whereas β1 and β3 integrins only mediate firm adhesion in physiological conditions [50]. It has been proposed that integrins in the extended conformation with low to intermediate affinity mediate cell rolling, whereas those with high affinity mediate firm adhesion [51]. Based on our experiments in this study, we propose that the β1 and β3 assume higher affinity for ligands because the MIDAS associated residue is Ala and not Asp, while the β2 and β7 integrins assume lower affinity. It is likely that during inside-out activation, conformation of integrins is shifted towards a more extended state. The β2 and β7 integrins in the extended conformation with lower affinity therefore support cell rolling, whereas the β1 and β3 integrins in the extended conformation with relatively high affinity adhere more strongly to ligands. This residue associated with the MIDAS may play a key role in integrin-mediated cell rolling and firm adhesion.

## Supporting Information

Figure S1 **The backbone heavy-atom root-mean-square deviations (RMSD) of the wild-type (WT) and mutant systems associated with two different divalent cation binding states at 300 K during the MD simulation courses.** The RMSD evolution indicates the degree of similarity of snapshots from MD simulations with the WT and mutant structures. (A) Mg2+-Ca2+-Ca2+, (B) Mn2+-Mn2+-Mn2+.
(PDF)

Figure S2 **The backbone atomic root-mean-square fluctuations (RMSF) values of the β I domain of integrin αIIbβ3 in wild-type (WT) and mutant forms against simulation time, which were obtained from the last 10 ns equilibrated MD trajectories.** The blue boxes indicate the regions of β-sheets while the purple boxes display the regions of α-helices, which are labeled to correspond with Arabic numerals. (A) Mg2+-Ca2+-Ca2+, (B) Mn2+-Mn2+-Mn2+.
(PDF)

Figure S3 **Ligand-protein interaction spectrum of five systems (a) wild-type (WT), (b) A252D, (c) A252D/D126A, (d) A252D/D127A, (e) A252D/D126A/D127A in Mg2+-Ca2+-Ca2+ state according to the MM/GBSA method.**
(PDF)

Figure S4 **Ligand-protein interaction spectrum of five systems (a) wild-type (WT), (b) A252D, (c) A252D/D126A, (d) A252D/D127A, (e) A252D/D126A/D127A in Mn2+-Mn2+-Mn2+ state according to the MM/GBSA method.**
(PDF)

Figure S5 **Key distances of the ions in the simulations with initially placed in Mg2+-Ca2+-Ca2+ state in wild-type (WT) and mutant forms during MD simulations.** Reported distances are as follows: (a) between MIDAS ions and the oxygen of Wat692, (b) between MIDAS ion and the oxygen of Wat756, (c) between MIDAS ion and the oxygen of Wat757, (d) between MIDAS ion and the oxygen of Wat758, (e) between MIDAS ion and the oxygen of Wat761, (f) between MIDAS ion and the oxygen of D119 (OD1), (g) between MIDAS ion and the oxygen of D119 (OD2), (h) between MIDAS ion and the oxygen of S121 (OG), (i) between MIDAS ion and the oxygen of S123 (OG), (j) between MIDAS ion and the oxygen of E220 (OE2), (k) between MIDAS ion and the RGD Asp carboxylic oxygen OD1, (l) between MIDAS ion and the RGD Asp carboxylic oxygen OD2, (m) between SyMBS ion and the oxygen of D158 (OD1), (n) between SyMBS ion and the oxygen of D158 (OD2), (o) between SyMBS ion and the oxygen of N215 (OD1), (p) between SyMBS ion and the oxygen of D217 (O), (q) between SyMBS ion and the oxygen of D217 (OD1), (r) between SyMBS ion and the oxygen of D217 (OD2), (s) between SyMBS ion and the oxygen of P219 (O), (t) between SyMBS ion and the oxygen of E220(OE1), (u) between ADMIDAS ion and the oxygen of S123 (O), (v) between ADMIDAS ion and the oxygen of D251 (OD2), (w) between ADMIDAS ion and the oxygen of D252 (OD1), (x) between ADMIDAS ion and the oxygen of M335 (O), (y) between MIDAS ion and SyMBS ion, (z) between MIDAS ion and ADMIDAS ion.
(PDF)

Table S1 **The contribution of energy for important individual amino acid residues towards the binding free energies computed by the MM/GBSA method (energies are in kcal·mol-1): (1) WT (2), A252D (3), A252D/D126A (4), A252D/D127A (5), A252D/D126A/D127A.**
(PDF)
