# Supplementary figures and images for: Variation in One Residue Associated with the Metal Ion-Dependent Adhesion Site Regulates αIIbβ3 Integrin Ligand Binding Affinity
Source: PLoS One. 2013 Oct 8;8(10):e76793. doi: 10.1371/journal.pone.0076793 (PMC3792891; doi:10.1371/journal.pone.0076793)

Figure S1

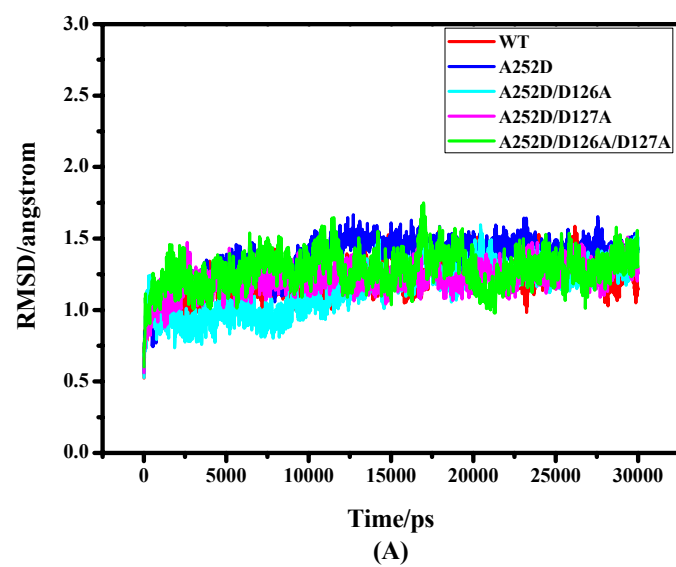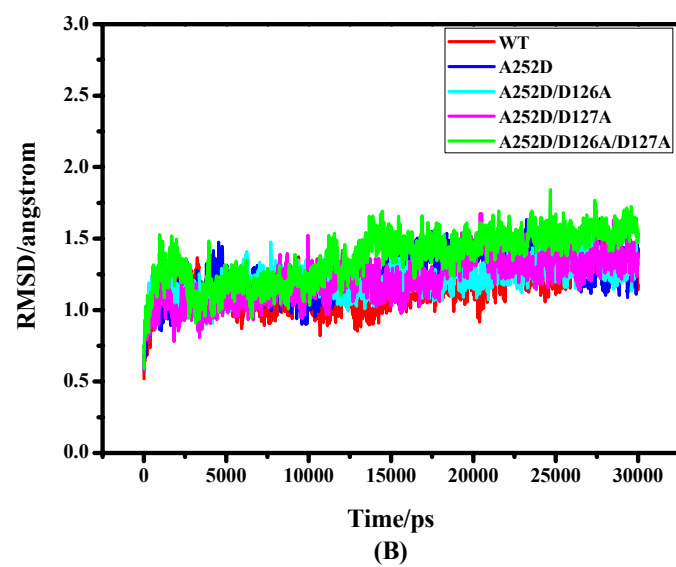

Supplement: Figure S1 — The backbone heavy-atom root-mean-square deviations (RMSD) of the wild-type (WT) and mutant systems associated with two different divalent cation binding states at 300 K during the MD simulation courses. The RMSD evolution indicates the degree of similarity of snapshots from MD simulations with the WT and mutant structures. (A) Mg2+-Ca2+-Ca2+, (B) Mn2+-Mn2+-Mn2+. (PDF) [file pone.0076793.s001.pdf]

Figure S2

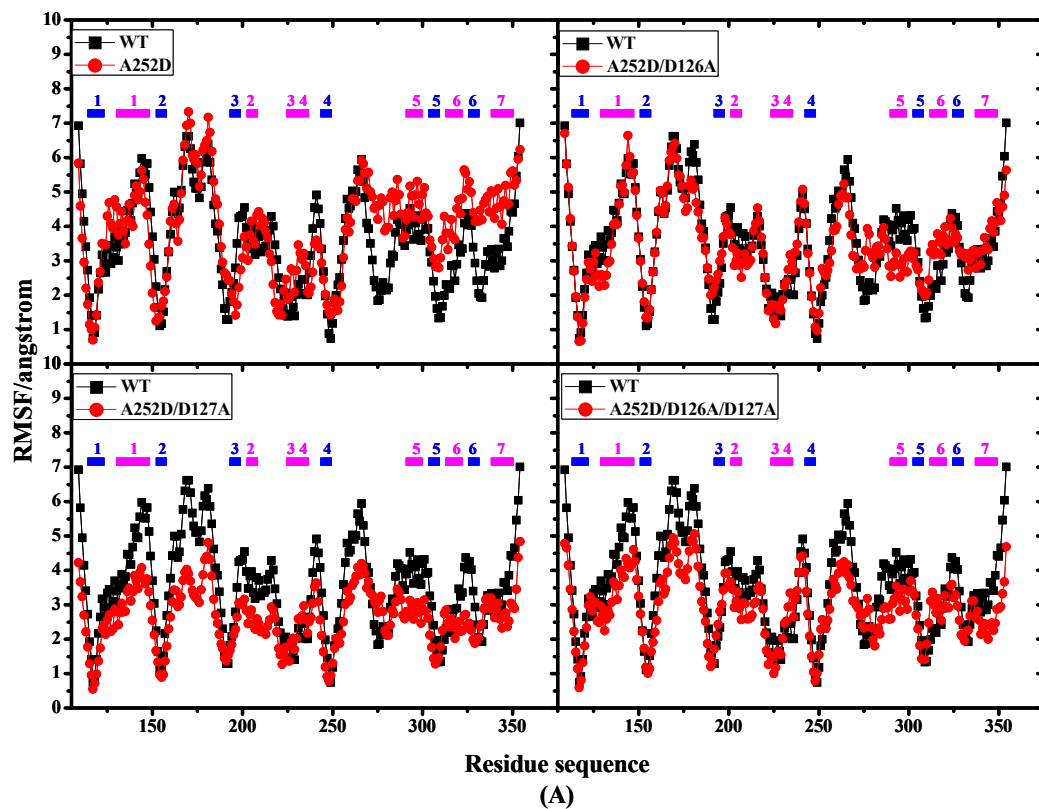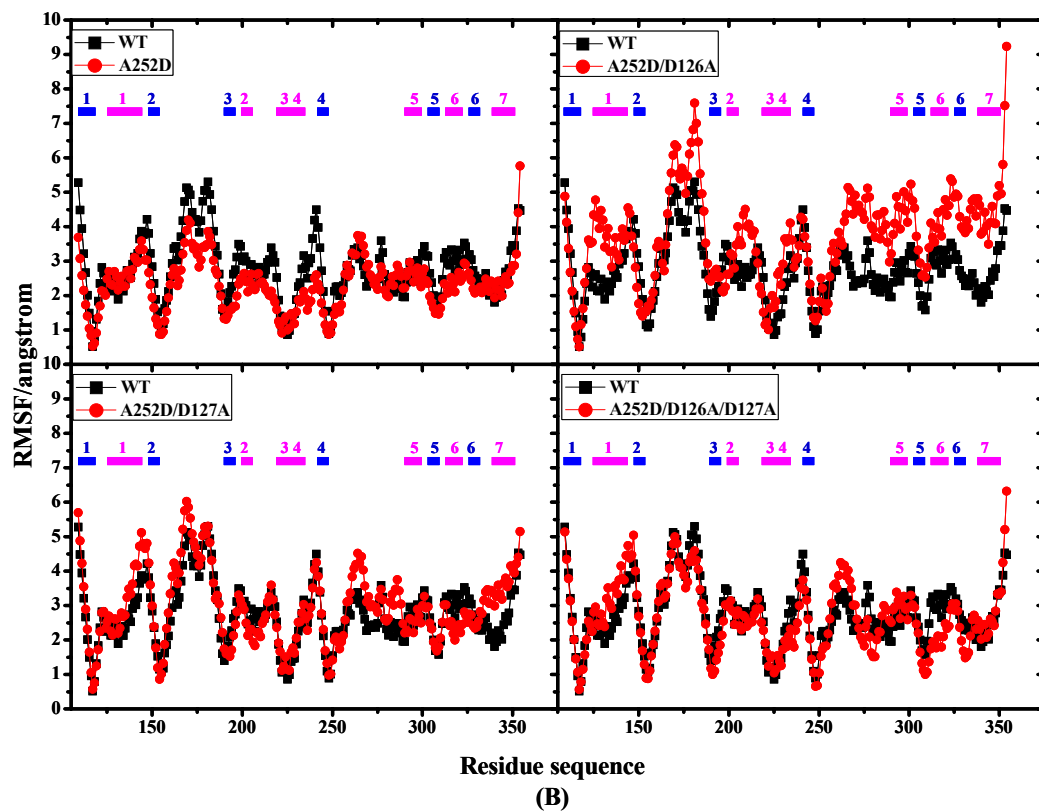

Supplement: Figure S2 — The backbone atomic root-mean-square fluctuations (RMSF) values of the β I domain of integrin αIIbβ3 in wild-type (WT) and mutant forms against simulation time, which were obtained from the last 10 ns equilibrated MD trajectories. The blue boxes indicate the regions of β-sheets while the purple boxes display the regions of α-helices, which are labeled to correspond with Arabic numerals. (A) Mg2+-Ca2+-Ca2+, (B) Mn2+-Mn2+-Mn2+. (PDF) [file pone.0076793.s002.pdf]

**Figure S3**

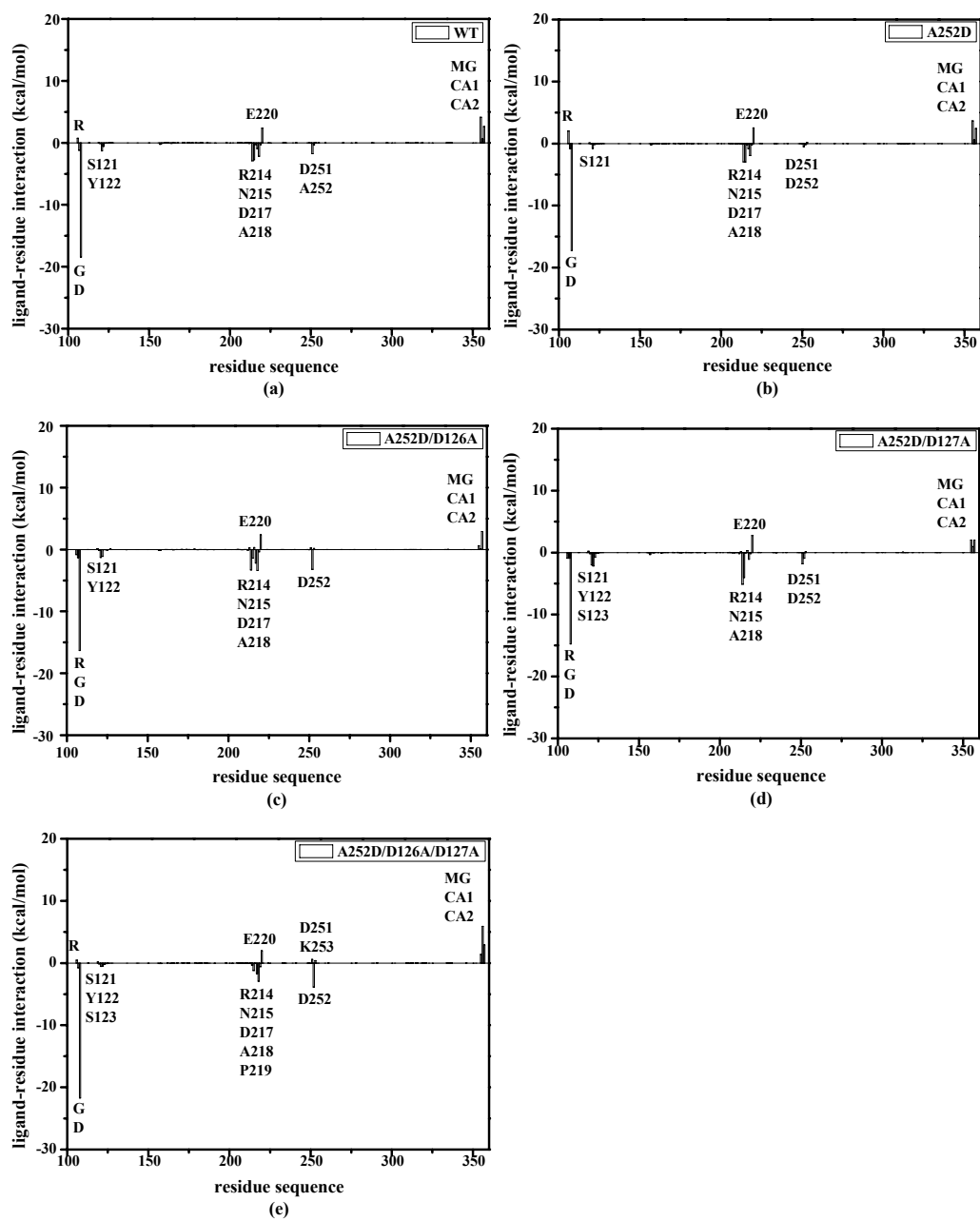

Supplement: Figure S3 — Ligand-protein interaction spectrum of five systems (a) wild-type (WT), (b) A252D, (c) A252D/D126A, (d) A252D/D127A, (e) A252D/D126A/D127A in Mg2+-Ca2+-Ca2+ state according to the MM/GBSA method. (PDF) [file pone.0076793.s003.pdf]

Figure S4

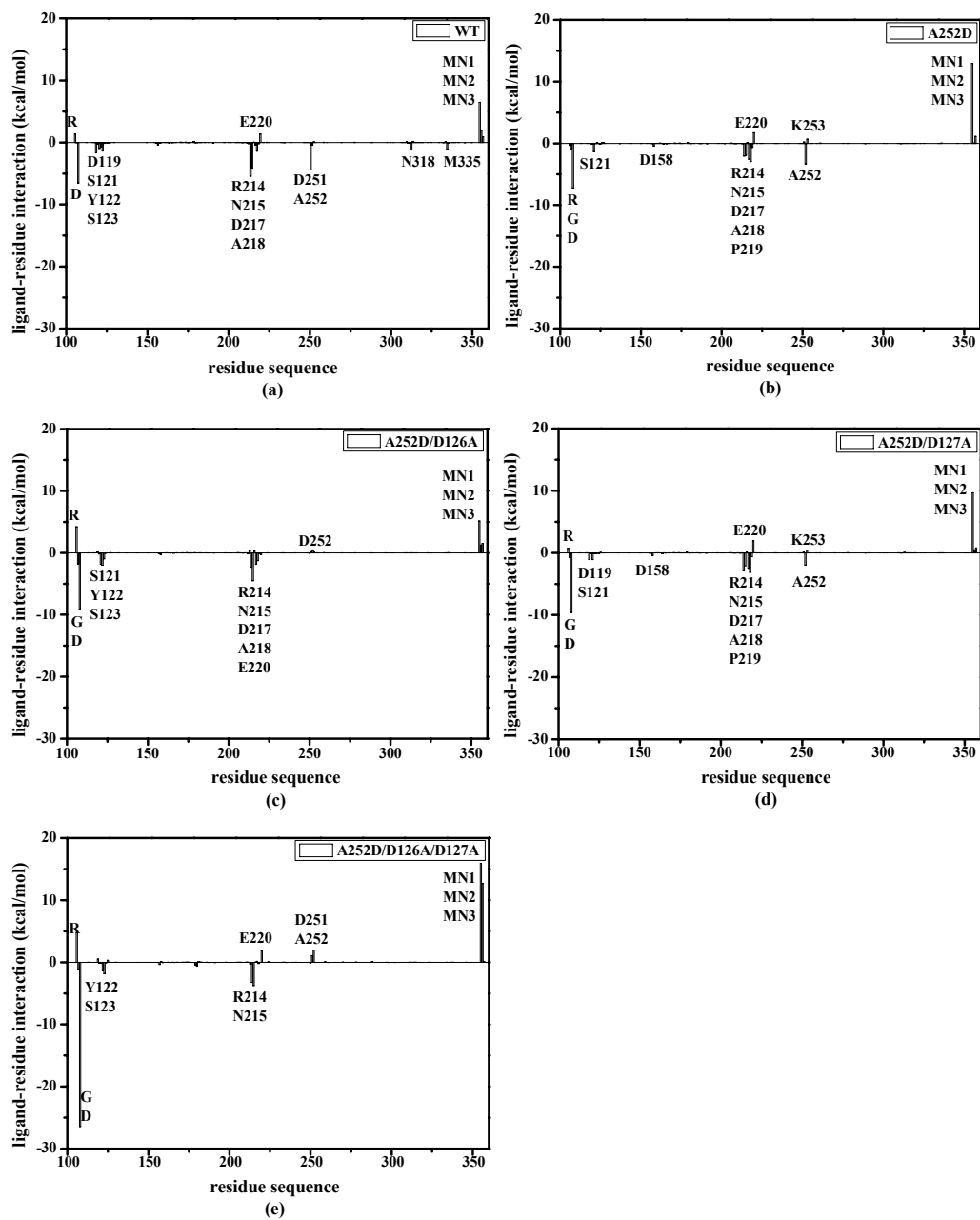

Supplement: Figure S4 — Ligand-protein interaction spectrum of five systems (a) wild-type (WT), (b) A252D, (c) A252D/D126A, (d) A252D/D127A, (e) A252D/D126A/D127A in Mn2+-Mn2+-Mn2+ state according to the MM/GBSA method. (PDF) [file pone.0076793.s004.pdf]

**Figure S5**

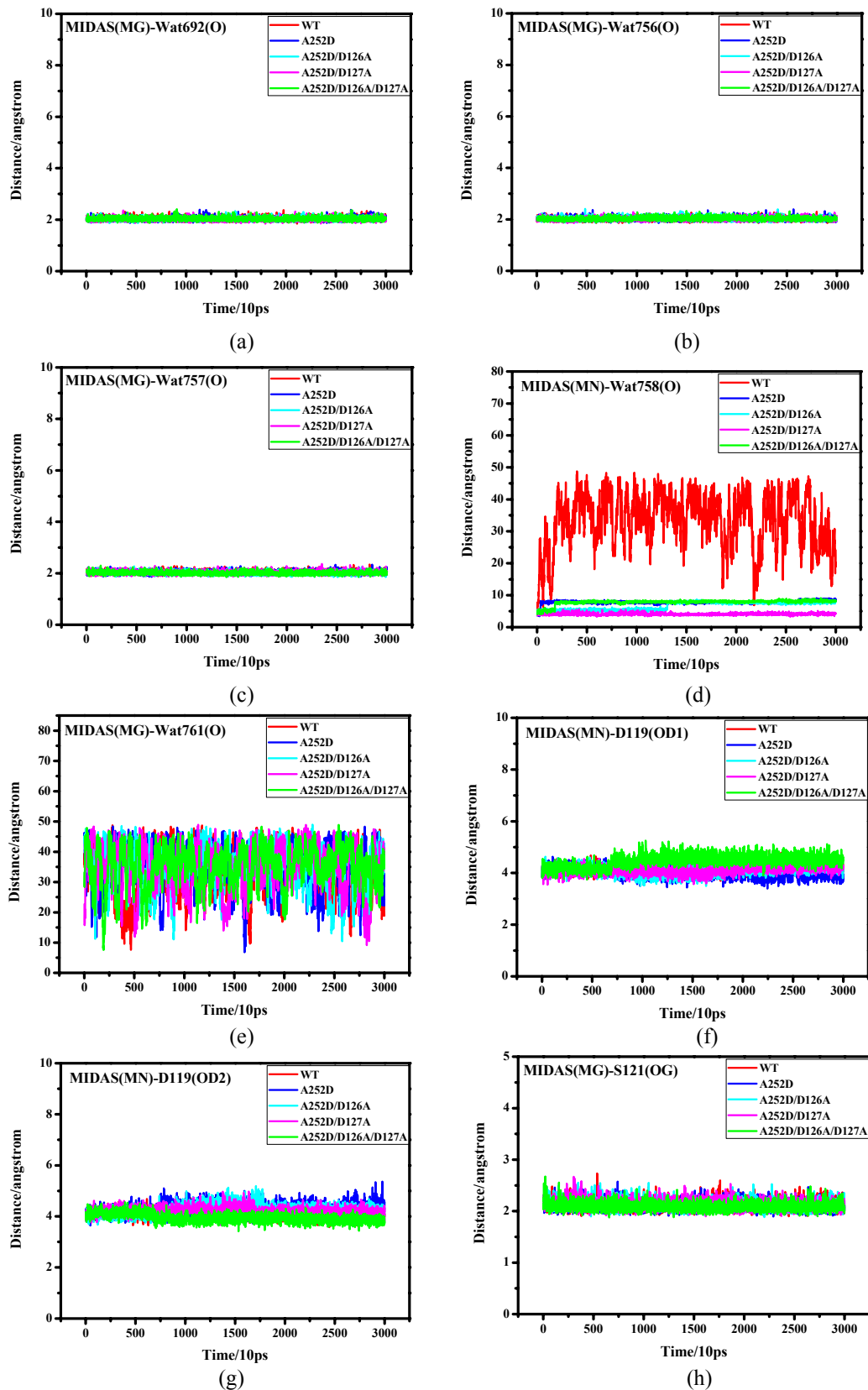

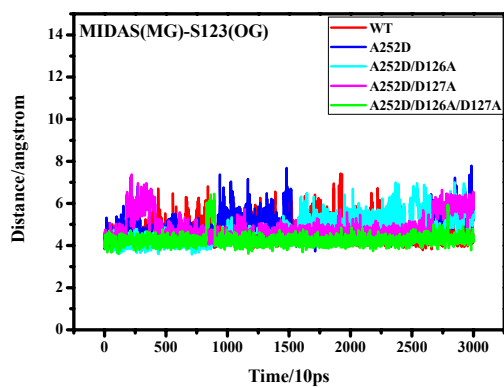

(i)

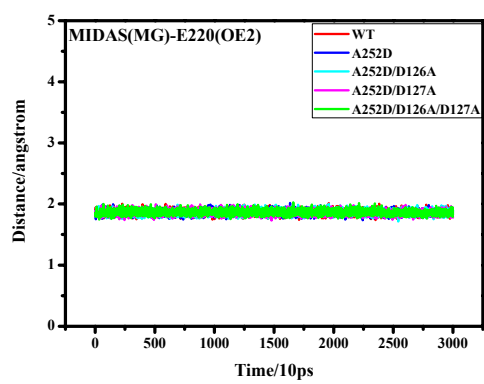

(j)

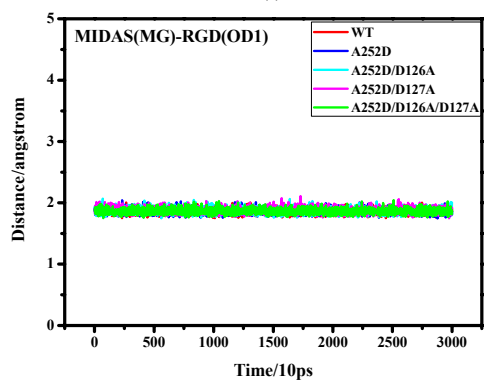

(k)

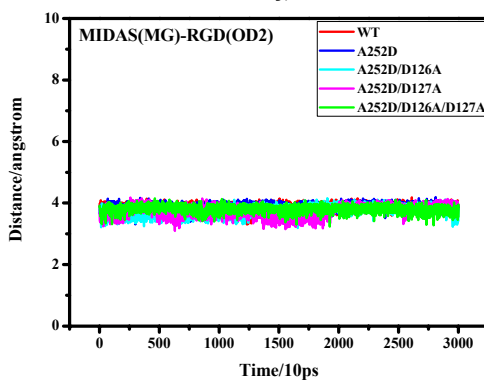

(l)

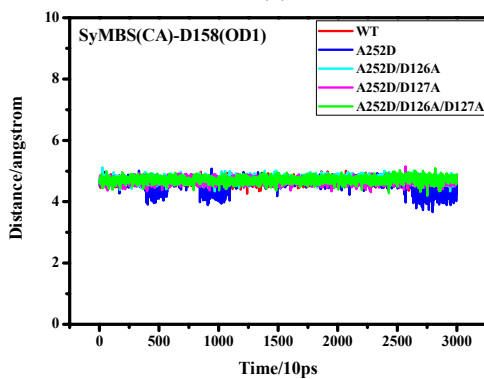

(m)

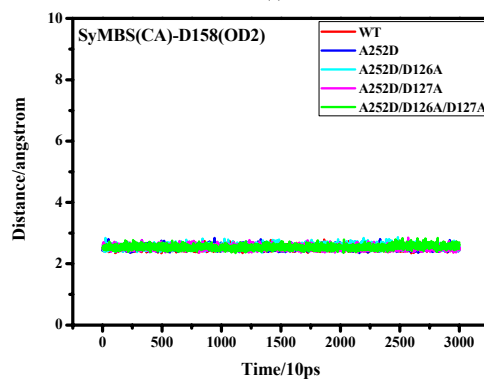

(n)

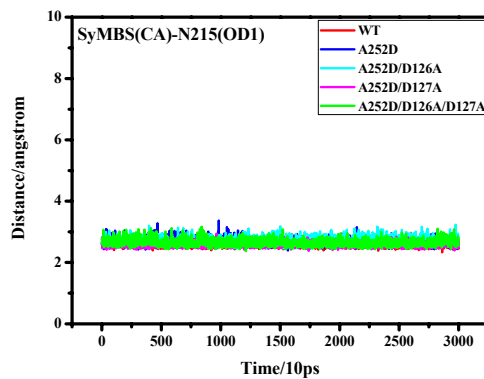

(o)

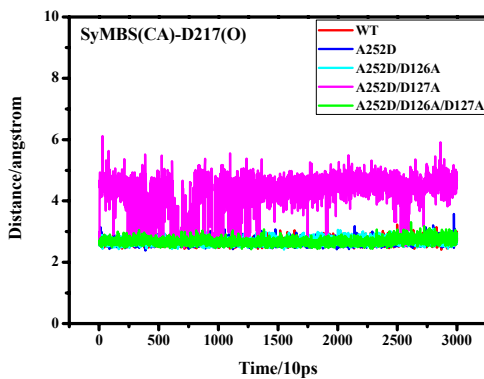

(p)

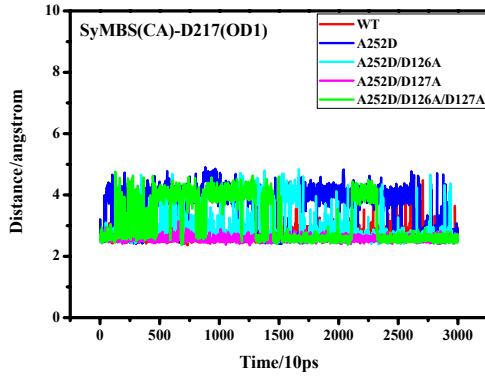

(q)

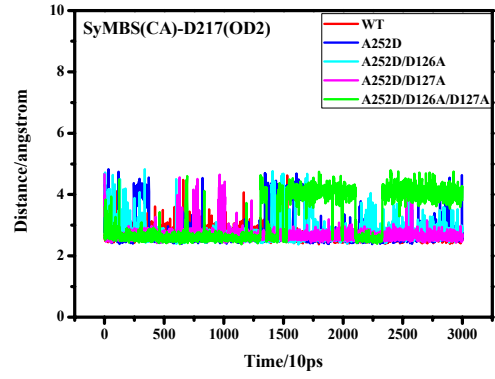

(r)

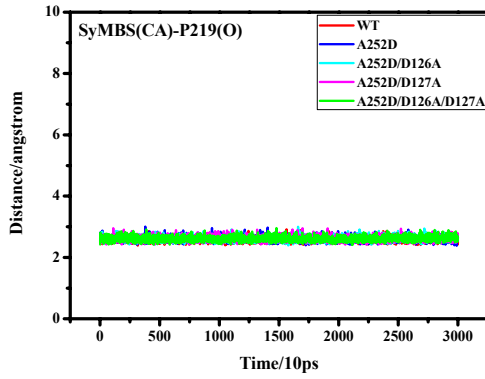

(s)

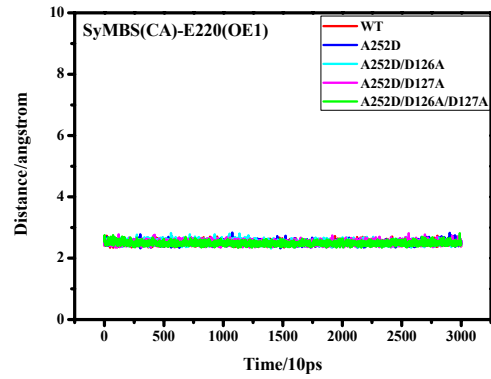

(t)

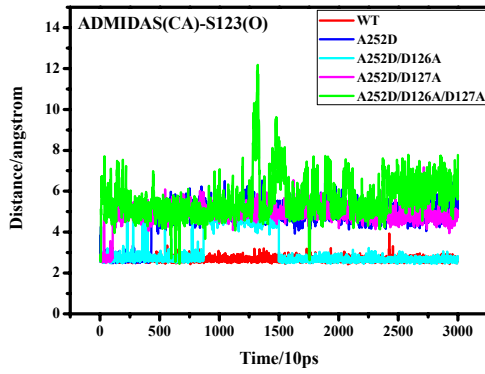

(u)

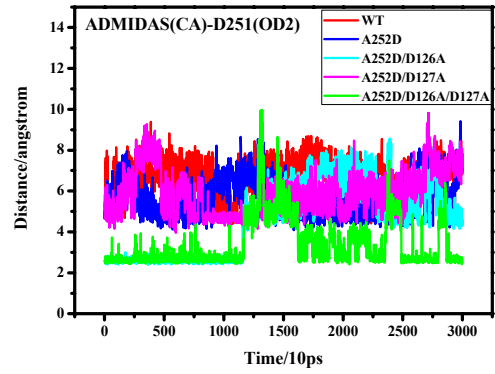

(v)

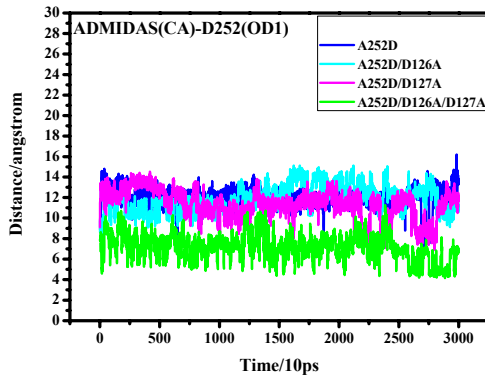

(w)

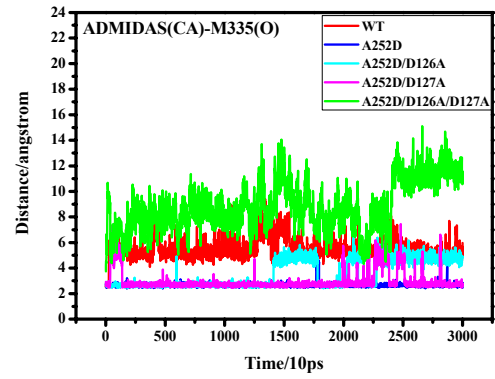

(x)

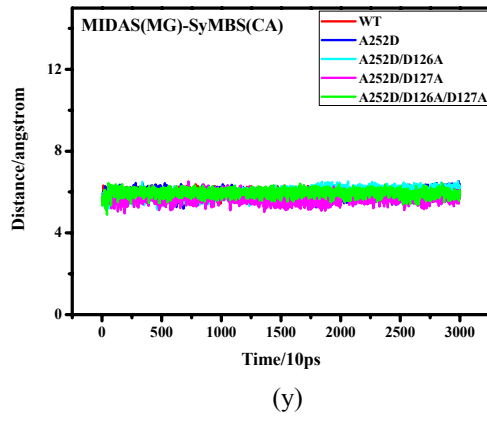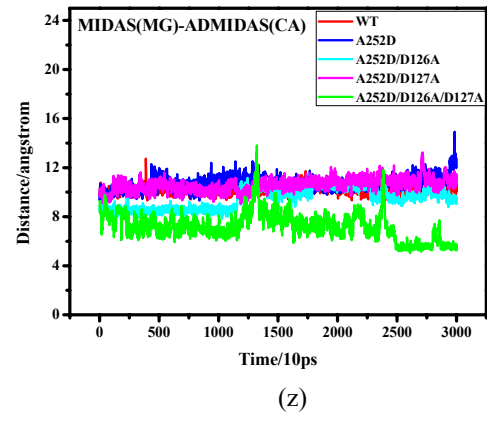

Supplement: Figure S5 — Key distances of the ions in the simulations with initially placed in Mg2+-Ca2+-Ca2+ state in wild-type (WT) and mutant forms during MD simulations. Reported distances are as follows: (a) between MIDAS ions and the oxygen of Wat692, (b) between MIDAS ion and the oxygen of Wat756, (c) between MIDAS ion and the oxygen of Wat757, (d) between MIDAS ion and the oxygen of Wat758, (e) between MIDAS ion and the oxygen of Wat761, (f) between MIDAS ion and the oxygen of D119 (OD1), (g) between MIDAS ion and the oxygen of D119 (OD2), (h) between MIDAS ion and the oxygen of S121 (OG), (i) between MIDAS ion and the oxygen of S123 (OG), (j) between MIDAS ion and the oxygen of E220 (OE2), (k) between MIDAS ion and the RGD Asp carboxylic oxygen OD1, (l) between MIDAS ion and the RGD Asp carboxylic oxygen OD2, (m) between SyMBS ion and the oxygen of D158 (OD1), (n) between SyMBS ion and the oxygen of D158 (OD2), (o) between SyMBS ion and the oxygen of N215 (OD1), (p) between SyMBS ion and the oxygen of D217 (O), (q) between SyMBS ion and the oxygen of D217 (OD1), (r) between SyMBS ion and the oxygen of D217 (OD2), (s) between SyMBS ion and the oxygen of P219 (O), (t) between SyMBS ion and the oxygen of E220(OE1), (u) between ADMIDAS ion and the oxygen of S123 (O), (v) between ADMIDAS ion and the oxygen of D251 (OD2), (w) between ADMIDAS ion and the oxygen of D252 (OD1), (x) between ADMIDAS ion and the oxygen of M335 (O), (y) between MIDAS ion and SyMBS ion, (z) between MIDAS ion and ADMIDAS ion. (PDF) [file pone.0076793.s005.pdf]
