# Supplementary material for: Variation in One Residue Associated with the Metal Ion-Dependent Adhesion Site Regulates αIIbβ3 Integrin Ligand Binding Affinity
Source: PLoS One. 2013 Oct 8;8(10):e76793. doi: 10.1371/journal.pone.0076793 (PMC3792891; doi:10.1371/journal.pone.0076793)
Supplement: Table S1 — The contribution of energy for important individual amino acid residues towards the binding free energies computed by the MM/GBSA method (energies are in kcal·mol-1): (1) WT (2), A252D (3), A252D/D126A (4), A252D/D127A (5), A252D/D126A/D127A. (PDF) [file pone.0076793.s006.pdf]

Supporting Information

Table S1 The contribution of energy for important individual amino acid residues towards the binding free energies computed by the MM/GBSA method (energies are in kcal·mol^{-1}): (1) WT, (2)A252D, (3) A252D/D126A, (4) A252D/D127A, (5) A252D/D126A/D127A.

	Mg^{2+}-Ca^{2+}-Ca^{2+}					Mn^{2+}-Mn^{2+}-Mn^{2+}				
**Residue**	**1**	**2**	**3**	**4**	**5**	**1**	**2**	**3**	**4**	**5**
Arg*	0.76	2.01	-0.83	-0.93	0.47	1.35	-0.34	4.24	0.73	4.74
Gly*	-1.2	-0.79	-1.32	-0.91	-0.78	-0.03	-0.94	-1.80	-0.77	-1.11
Asp*	-18.45	-17.27	-16.35	-14.76	-21.71	-6.54	-7.24	-9.18	-9.61	-26.47
Asp^{119}	0.08	0.14	0.12	0.23	0.16	-1.69	-0.06	0.14	-1.06	0.51
Ser^{121}	-1.30	-0.81	-1.29	-2.00	-0.58	-0.97	-1.36	-1.85	-1.10	-0.13
Tyr^{122}	-0.56	-0.17	-1.12	-2.17	-0.47	-0.74	-0.04	-2.01	-0.13	-1.34
Ser^{123}	-0.06	-0.13	-0.05	-0.75	-0.24	-1.38	0.10	-0.99	-0.10	-1.83
Asp^{158}	-0.09	-0.04	-0.11	0.01	-0.10	-0.11	-0.45	-0.27	-0.43	0.11
Asp^{179}	0.07	0.07	0.09	0.06	0.09	0.19	0.12	0.04	0.11	-0.44
Met^{180}	-0.01	-0.02	-0.01	-0.01	-0.01	-0.05	-0.01	-0.02	-0.01	-0.63
Arg^{214}	-2.92	-2.98	-3.33	-5.13	-0.33	-5.41	-2.05	-2.32	-2.88	-3.26
Asn^{215}	-2.72	-3.00	-1.40	-4.05	-1.21	-4.13	-1.95	-4.52	-2.09	-3.75
Asp^{217}	-0.95	-0.82	-2.21	0.30	-1.71	-0.39	-2.60	-1.85	-2.50	0.15
Ala^{218}	-2.18	-1.93	-3.38	-1.08	-2.95	-1.42	-2.98	-1.28	-3.13	-0.23
Pro^{219}	-0.29	-0.21	-0.40	-0.20	-0.60	-0.21	-0.66	-0.17	-0.60	-0.05
Glu^{220}	2.38	2.54	2.39	2.78	1.98	1.35	1.75	-0.31	1.99	1.84
Asp^{251}	-1.73	-0.52	0.26	-1.80	0.61	-4.42	0.22	0.18	0.12	1.07
Ala^{252}/Asp^{252}	-0.33	-0.18	-3.21	-0.93	-3.88	-0.38	-3.36	0.36	-1.96	1.98
Lys^{253}	0.08	0.20	0.18	0.16	0.41	0.11	0.69	0.08	0.45	-0.05
Asn^{313}	-0.01	-0.03	0.01	0.07	0.06	-1.22	0.03	0.03	0.08	-0.02
Met^{335}	0.01	0.00	0.01	-0.01	0.01	-1.12	0.01	0.01	0.01	-0.02
MIDAS	4.15	3.68	0.63	2.05	1.38	6.48	12.95	5.16	9.68	15.92
ADMIDAS	0.62	0.61	0.13	0.98	5.90	1.99	0.05	1.24	0.50	12.68
SyMBS	2.64	2.45	2.90	2.03	2.92	0.93	1.12	1.47	0.82	0.07

* represents Ligand RGD
